# Application for simulating public health problems during floods around the Loei River in Thailand: the implementation of a geographic information system and structural equation model

**DOI:** 10.1186/s12889-022-14018-7

**Published:** 2022-08-31

**Authors:** Tanunchai Boonnuk, Kirati Poomphakwaen, Natchareeya Kumyoung

**Affiliations:** Public Health Program, Department of Applied Science, Faculty of Science and Technology, Loei Rajabhat University, Loei, 42000 Thailand

**Keywords:** Flood disaster, Structural equation model, Geographic information system

## Abstract

**Background:**

Floods cause not only damage but also public health issues. Developing an application to simulate public health problems during floods around the Loei River by implementing geographic information system (GIS) and structural equation model (SEM) techniques could help improve preparedness and aid plans in response to such problems in general and at the subdistrict level. As a result, the effects of public health problems would be physically and mentally less severe.

**Methods:**

This research and development study examines cross-sectional survey data. Data on demographics, flood severity, preparedness, help, and public health problems during floods were collected using a five-part questionnaire. Calculated from the population proportion living within 300 m of the Loei River, the sample size was 560 people. The participants in each subdistrict were recruited proportionally in line with the course of the Loei River. Compared to the empirical data, the data analysis examined the causal model of public health problems during floods, flood severity, preparedness, and help. The standardized factor loadings obtained from the SEM analysis were substituted as the loadings in the equations for simulating public health problems during floods.

**Results:**

The results revealed that the causal model of public health problems during floods, flood severity, preparation, and help agreed with the empirical data. Flood severity, preparedness, and aid **(**χ^2^ = 479.757, df = 160, *p* value <.05, CFI = 0.985, RMSEA = 0.060, χ^2^/df = 2.998) could explain 7.7% of public health problems. The computed values were applied in a GIS environment to simulate public health problem situations at the province, district, and subdistrict levels.

**Conclusions:**

Flood severity and public health problems during floods were positively correlated; in contrast, preparedness and help showed an inverse relationship with public health problems. A total of 7.7% of the variance in public health problems during floods could be predicted. The analysed data were assigned in the GIS environment in the developed application to simulate public health problem situations during floods.

**Supplementary Information:**

The online version contains supplementary material available at 10.1186/s12889-022-14018-7.

## Background

Flooding is a major problem worldwide. A few examples include floods in the Mississippi basin [1] and the Amazon River basin [2] in the Americas, floods in the Danube River basin in Europe [3], floods in the Nile basin in Africa [4], floods in the Yangtze River basin [5] and the Mekong River [6, 7] in Asia. Thailand also frequently deals with flooding. There have been several major floods in the country, for instance, flash floods and landslides in Wang Chin district, Phrae Province, and in Lom Sak district, Phetchabun Province, in 2001 [8]; in Laplae district, Tha Pla district, and Mueang district in Uttaradit Province in 2006 [9]; and massive floods in the central plain in 2011 [10]. The occurrence of flooding in 2011 became more frequent and more severe over time [11]. Floods can have severe impacts on large areas, such as agricultural areas, industrial estates, commercial districts, and residential areas, in several regions, including Bangkok. According to reports of provinces affected by floods in Thailand, 4,405,315 people from 1,590,346 households were affected by the end of 2011 [12]. In Loei Province, due to overflow from the Loei River, four floods in 2017 damaged the vicinity and caused fatalities [13]. Flooding in Loei Province exerts an enormous impact on the lives of the people who reside in the riverside area. Because the Loei River originates in the Phu Luang mountain area, any additional, unexpected water flow can result in rapid flooding. Furthermore, water management in the dams upstream of the Loei River and the tributaries that flow into the Loei River is affected by considerable water storage throughout the rainy season to prepare for sustaining agriculture, which is the main occupation of the population, throughout the summer drought. This additional water retained in the dam could cause erosion damage, thereby necessitating accelerated drainage to prevent erosion. This drainage, combined with the accelerated release of water from 14 branch reservoirs, results in the repeated flooding of houses in the river area. Such floods last approximately 2 days because the water ultimately flows into the Mekong River, where the water level is already high due to the rainy season and considerable water flowing in from China. As a result, water drains from the Loei area quite slowly, and the flooding of houses during this period results in negative consequences including electrical accidents due to downed wires, increased encounters with dangerous animals such as snakes and scorpions, disease outbreaks, food shortages and mental health problems. Demographically, most people in the river basin area live in rural societies. Geographically, the area is a plain surrounded by mountains. In Thailand, the administrative characteristics of this area are central (district, province, region, and country levels) and local (subdistrict level). There are two types of governance at the subdistrict level: municipalities (in urban areas) and subdistrict administrative organizations (in rural areas). The subdistrict administrative organization responsible for almost all of the Loei River Valley subdistrict also takes partial responsibility for managing flood problems. Both the government and public sector also take responsibility for flood issues through a collaboration of many departments, including government agencies, public health agencies, and disaster mitigation agencies. The public sector provides volunteer rescue services. These two components form an ad hoc working group for the management of flood-related disasters.

The negative aftermath of disastrous floods can affect the economy, society, and the environment [14]. Some consequences are, for instance, destruction or damage to houses and buildings, loss of lives and animals, and epidemics [15]. Floods can also result in food and water shortages [16]. These flood consequences can lead to public health problems, including epidemics, such as cholera, leptospirosis, hepatitis, and diseases caused by animals and insects, and mental health problems, such as anxiety disorder and depression, especially among the elderly [17]. Moreover, floods also obstruct the transportation needed to receive health services, particularly for patients who require continuous care.

In recent decades, there has been a trend to use more advanced data analysis techniques in research studies to answer research questions, including structural equation modelling analysis. The structural equation model (SEM) is a statistical method for investigating the correlations between variables. It can measure a relationship between observed and latent variables or between two or more latent variables. Compared with regression analysis, SEM analysis is more advantageous for researchers in terms of flexibility. It allows relationships between several predictor variables (creating a latent variable that is unable to be measured directly), errors in the measurement of observed variables, and statistical tests between hypotheses and empirical data [18]. Several studies have applied the SEM technique to analyse flood issues [19–21].

A geographic information system (GIS) is a computer information system used to import, manage, analyse, and export geographic data. It can gather, store, fetch, manage, analyse data and exhibit spatial correlations [22], relying on geographical features to link datasets and reveal correlations. The results are usually presented in a map displaying spatial data with distributions based on the area of interest. Many research studies have also implemented GIS to analyse flood situations [23–25]. Some have used GIS to simulate flood situations [26, 27] and applied a regression equation to colour the map [28].

Since floods can cause considerable damage and public health problems, a situation simulator should be developed and utilized for preparation and aid plans. The capabilities of GIS can be used to help clearly simulate situations. Previous studies have adopted regression equations and GIS to simulate situations; however, regression equations have various analytical limitations. Therefore, the researchers in this study would like to introduce a solution by implementing both SEM and GIS techniques to improve the simulations.

The objectives of this study are to investigate the causal model among public health problems during floods, flood severity, preparation, and help and to develop an application with SEM and GIS to simulate public health problems around the Loei River during floods. Further explanations are provided in the next section.

## Methods

### Conceptual framework

**Fig. 1:**
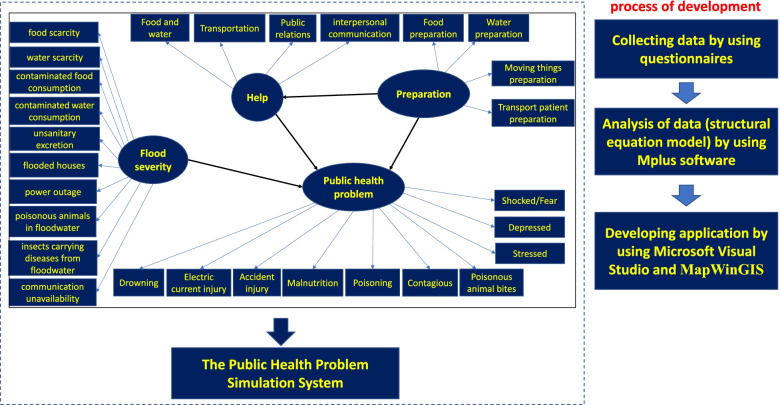
Conceptual framework and application development process

This research is a cross-sectional study, the research results of which will be used in the development of further applications. This cross-sectional study involves research and development with two objectives: 1) to investigate the causal model among flood severity, preparedness, help, and public health problems during floods and 2) to develop an application to simulate public health problem situations around the Loei River during floods using GIS and SEM. The disaster management guidelines for flood mitigation, involving prevention, preparation, response, and help, were focused on when creating the SEM [29]. Apart from reducing the severity of public health problems, prevention and preparation plans can also improve response and assistance. For that reason, the conceptual framework and application development process is shown in Fig. 1 below.

### Data collection

#### Population and sample size

The population in this study included the people residing within 300 m of the Loei River Basin. Participants were recruited from 35 subdistricts located near the Loei River. The number of participants in each subdistrict was proportional based on the distance from the river. The sample was obtained through simple random sampling of households near the Loei River within 300 m of each subdistrict. Proportional sampling from each subdistrict was calculated by selecting a representative from each household to serve as an informant who could remember as many details as possible about flood incidents. The sample size was approximately 20 times greater than the number of observed variables [30]. There were 28 observed variables; hence, the sample size was 560 people (28 observed variables multiplied by 20 (28*20 = 560 people)). The data of the respondents from each subdistrict were collected corresponding to the course of the Loei River.

#### Research instrument

The instrument used in this study was a questionnaire consisting of five parts as follows: 1) a checklist of demographic questions about gender, age, marital status, income, and the number of household members; 2) questions about direct problems from floods (ten items); 3) questions about preparedness (four items); 4) questions about aid (four items); and 5) questions about public health problems during floods (ten items). Parts two to five were a 0-to-10 rating scale with 11 rating choices for each item. The validity of the questionnaire was evaluated by a disaster management expert, a GIS expert, a local disaster management official, a public health officer specializing in disaster management, and an independent disaster management scholar. The IOC value was higher than 0.5; however, the questionnaire was revised following the experts’ suggestions. The revised questionnaire was piloted with the people living in a river basin in Nong Bua Lamphu Province, and the improved IOC value was higher than 0.7.

#### Ethics and data collection

1. The research proposal and instrument were submitted to the Research Ethics Committee of Loei Rajabhat University for the certificate of approval.
2. For the research instrument tryout, 30 copies of the questionnaire were distributed to the respondents in a river basin in Nong Bua Lamphu Province. After the quality assessment, the questionnaire was revised. Questionnaires were created based on the researcher’s literature review (reliability values were checked to ensure that they met the requirements).
3. For data collection, the researchers and research assistants distributed 580 copies of the questionnaire to the respondents in person. The respondents were informed about the research objectives and the protection of their rights.
4. The returned questionnaire copies were checked for any missing data before the data were imported for later analysis.
5. The data collection occurred from July 1, 2020, until June 30, 2021.

### Data analysis

1. Descriptive statistics were used to analyse the data of respondents’ demographic information. Frequency and percentage metrics are used for the qualitative data. For the quantitative data, if normally distributed, means and standard deviation are presented, whereas the median, maximum, and minimum are shown in case of nonnormal distributions.
2. Mplus version 7.4 was used for structural equation modelling to examine the causal model among flood severity, preparation, help, and public health problems during floods compared with the empirical data.

### The development of an application simulating public health problem situations during floods

To create a system to simulate public health problems during floods, the standardized factor loadings from structural equation modelling acted as loadings for computing the scores of public health problems during floods. The following equations were used for the score calculation.

1 $$\mathbf{public}\ \mathbf{health}\ \mathbf{problem}\ \mathbf{score}=\mathbf{direct}\ \mathbf{score}+\mathbf{indirect}\ \mathbf{score}$$

In terms of score calculation, when each variable’s standardized factor loading, ranging from zero to ten, was available and the scores of public health problems were between zero and ten, normalization was applied as follows:

2 $$\mathrm{S}=\frac{\left({C}_1F+{C}_2H+{C}_3P\right)+\left({C}_2{C}_4 HP\right)}{\left({C}_1+{C}_2+{C}_3+10\left({C}_2{C}_4\right)\right)}$$

where *S* stands for the score of public health problems

*F*stands for flood severity

*H*stands for help (flood relief)

*P*stands for preparation (preparedness)

C_1_stands for the standardized factor loading from severity to public health problems

C_2_stands for the standardized factor loading from help to public health problems

C_3_stands for the standardized factor loading from preparedness to public health problems

C_4_stands for the standardized factor loading from preparedness to help

Help and preparation were the factors opposing public health problems. While the maximum and minimum scores of flood severity, help, and preparation ranged from zero to ten, the C_2_ and C_3_ standardized factor loadings were negative due to being opposing factors. Hence, the equation was adjusted to Eq. (3) below.

3 $$\mathrm{S}=\frac{C_1F+\left|{C}_2\right|\left(10-H\right)+\left|{C}_3\right|\left(10-P\right)+\left|{C}_2{C}_4\right|\left(10-H\right)\left(10-P\right)}{C_1+\left|{C}_2\right|+\left|{C}_3\right|+10\left|{C}_2{C}_4\right|}$$

For the worst case, the values of the most severe flood (F = 10), no help (*H* = 0), and no preparation (*P* = 0) were substituted in Eq. (3), and the severity score was highest (S = 10), as shown in Eq. (4).

4 $$\mathrm{S}=\frac{10{C}_1+10\left|{C}_2\right|+10\left|{C}_3\right|+100\left|{C}_2{C}_4\right|}{C_1+\left|{C}_2\right|+\left|{C}_3\right|+10\left|{C}_2{C}_4\right|}=\frac{10\left({C}_1+\left|{C}_2\right|+\left|{C}_3\right|+10\left|{C}_2{C}_4\right|\right)}{C_1+\left|{C}_2\right|+\left|{C}_3\right|+10\left|{C}_2{C}_4\right|}=10$$

For the best case, the values of the least severe flood (F = 0), great help (*H* = 10), and great preparation (*P* = 10) were substituted in Eq. (3), and the severity score was the lowest (S = 0), as shown in Eq. (5):

5 $$\mathrm{S}=\frac{0{C}_1+0\left|{C}_2\right|+0\left|{C}_3\right|+0\left|{C}_2{C}_4\right|}{C_1+\left|{C}_2\right|+\left|{C}_3\right|+10\left|{C}_2{C}_4\right|}=\frac{0}{C_1+\left|{C}_2\right|+\left|{C}_3\right|+10\left|{C}_2{C}_4\right|}=0$$

**Fig. 2:**
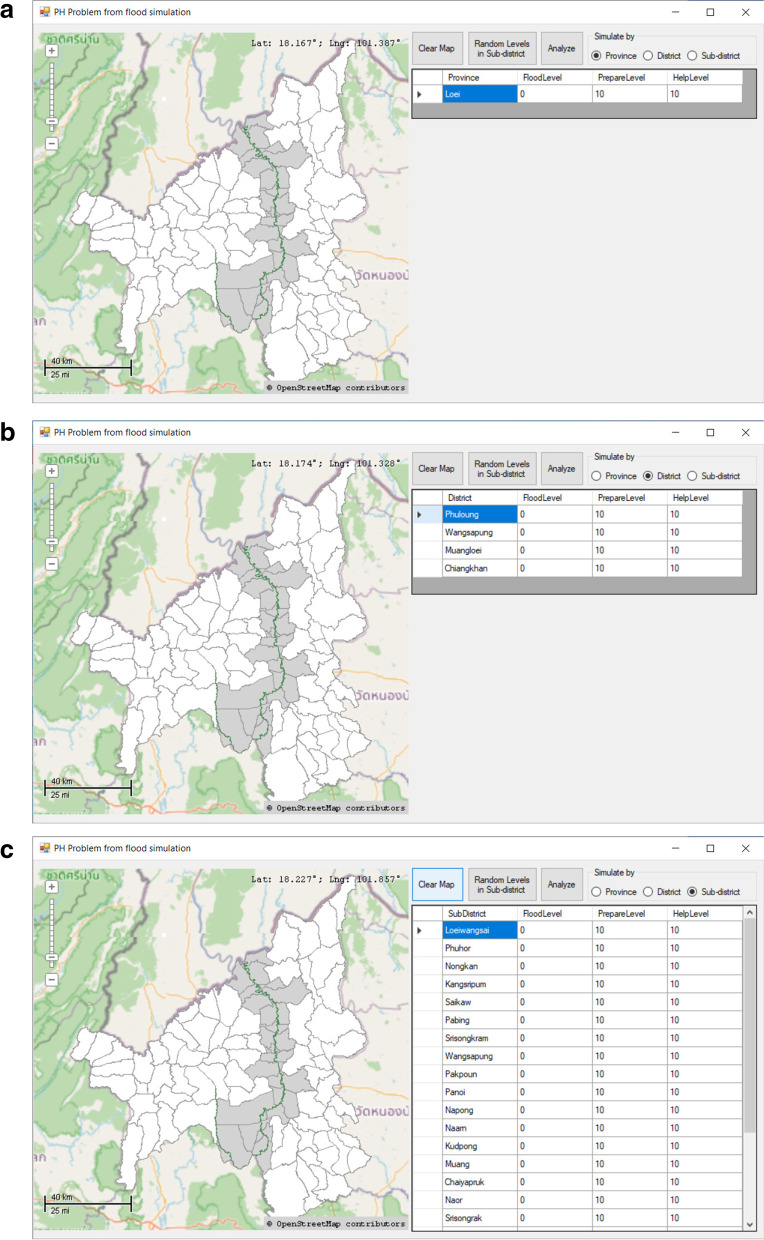
Screenshots from the application simulating public health problems during floods

The application was developed with Visual Studio 2017. Additionally, MapWinGIS version 5.3.0 was also used for map generation. Screenshots of the application can be seen in Fig. 2 below.

This simulation will assist both with preventive planning and when a public health problem arises. When flooding occurs, issues can arise at both the district and provincial levels, especially when part of the flooded area is at the subdistrict level, because preparation and assistance involve both manpower and budget. If such efforts are overprepared, the area may experience budget and manpower losses that then affect other elements such as education and road development. In contrast, if too little effort is made in these areas, public health problems caused by flooding may not be resolved in a timely manner or may escalate to a higher level, such as an outbreak of water-borne diseases or loss of life and property. The simulation helps predict the level of the problem and determine the most appropriate level of preparation and assistance to most effectively reduce the occurrence of public health problems.

## Results

The results were divided into two parts based upon the research objectives.

### Structural equation model analysis

#### The analysis of demographic information

The demographic information analysis revealed that most of the respondents were female (62.9%), aged 35–59 (46.1%) ($\overline{x}$ = 53.23, SD = 16.51), married (85.2%), elementary school graduates (71.1%), farmers (53.2%), earned between 1001 and 10,000 baht per month (62.3%) (Median = 3000, Max = 60,000, Min = 0) and had 4–6 household members (64.3%) ($\overline{x}$ = 4.79, SD = 1.66). The details are displayed in Table 1.

**Table 1:** Respondents’ demographic information

Demographic information	Number of respondents (***n*** = 560)	Percentage
**Gender**		
Male	208	37.1
Female	352	62.9
**Age**		
Under 35 years old	83	14.8
35–59 years old	258	46.1
60 years old and over	219	39.1
$\overline{x}$ = 53.23, SD = 16.51		
**Marital status**		
Married	477	85.2
Single	70	12.5
Widowed/divorced/separated	13	2.3
**Education**		
None	20	3.5
Elementary	398	71.1
High school	113	20.2
Diploma/Bachelor’s degree	28	5.0
Master’s degree or higher	1	0.2
**Occupation**		
Farmer	298	53.2
Unemployed	100	17.9
Freelancer	68	12.1
Merchant/vender	68	12.1
Civil servant	9	1.6
Others	17	3.1
**Average monthly income**		
No income	47	8.4
Less than 1000 Baht	115	20.5
1001–10,000 Baht	349	62.3
More than 10,000 Baht	49	8.8
Median = 3000, Max = 60,000, Min = 0		
**Number of household member(s)**		
1–3 member(s)	118	21.1
4–6 members	360	64.3
7 members or over	82	14.6
$\overline{x}$ = 4.79, SD = 1.66		

#### Analysis of the causal model including flood severity, preparation, help, and public health problems during floods with empirical data

**Fig. 3:**
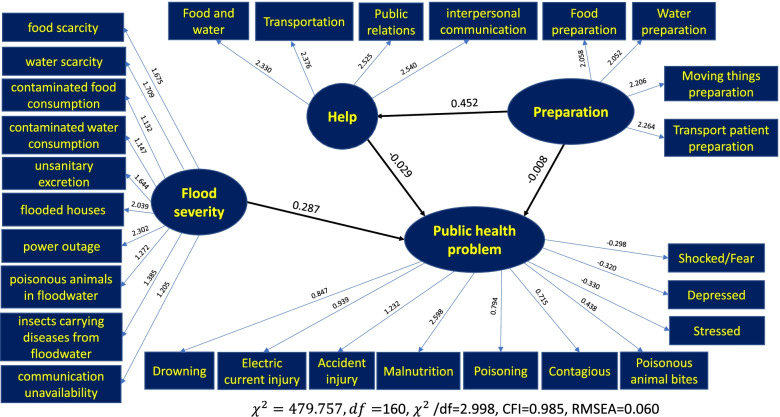
Causal model including flood severity, preparation, help, and public health problems during floods

The SEM was adjusted as per the fit index to examine the causal model. After the adjustment, the model became fit with the empirical data considering the following statistics used for the model’s validity test: χ^2^ = 479.757, df = 160, *p* value <.05, CFI = 0.985, RMSEA = 0.060, and χ^2^/df = 2.998, which was fit with the empirical data being lower than three [31]. A CFI value greater than 0.9 indicates a good level of fit [32]. An RMSEA value less than 0.08 [33] is also within the acceptable standard; hence, the model matched the empirical data. These analysis results led to acceptance of the hypothesis that the causal model among flood severity, preparation, help, and public health problems agreed with the empirical data. Additionally, the severity, preparation, and help were able to simulate situations of public health problems during floods by 7.7%, as shown in Fig. 3 and Table 2.

**Table 2:** The analysis results of the effect values between independent and dependent variables

**Independent variables**	**Dependent variables**					
	Help			Public health problems		
	TE	IE	DE	TE	IE	DE
Flood severity	–	–	–	0.287	–	0.287
Preparation	0.452	–	0.452	−0.021	−0.013	−0.008
Help	–	–	–	−0.029	–	−0.029
**Statistical values**	*χ*^2^= 479.757	df = 160	*p* value < .05	*χ*^2^/df = 2.998	CFI = .985	RMSEA = 0.060
**Structural equation**	Help			Public Health problems		
Square Multiple Correlation	20.5%			7.7%		

### Testing the system for simulating public health problem situations during floods

The standardized factor loadings from the structural equation modelling analysis were substituted into Eq. (3) as shown in the equations below.

6 $$\mathrm{S}=\frac{0.287F+\left|-0.029\right|\left(10-H\right)+\left|-0.008\right|\left(10-P\right)+\left|\left(-0.029\right)(0.452)\right|\left(10-H\right)\left(10-P\right)}{0.287+\left|-0.029\right|+\left|-0.008\right|+10\left|\left(-0.029\right)(0.452)\right|}\kern2.75em$$

7 $$\mathrm{S}=\frac{0.287F+0.029\left(10-H\right)+0.008\left(10-P\right)+0.013108\left(10-H\right)\left(10-P\right)}{0.287+0.029+0.008+(10)(0.013108)}$$

8 $$\mathrm{S}=\frac{0.287F+0.029(10)-0.029H+0.008(10)-0.008P+0.013108\left(100-10H-10P+ HP\right)}{0.287+0.029+0.008+0.13108}$$

9 $$\mathrm{S}=\frac{0.287F+0.29-0.029H+0.08-0.008P+1.3108-0.13108H-0.13108P+0.013108 HP}{0.45508}$$

10 $$\mathrm{S}=\frac{0.287F-0.18908H-0.13908P+0.013108 HP+1.6808}{0.45508}$$

With Eq. (10), the rating scale points 0–10 were substituted in every case possible. The total number of cases (11x11x11) was 1331. The testing of the computed values showed a nonnormal distribution. For that reason, the data of values were separated into 11 ranks by percentiles. The acquired values were translated into 11 levels of public health problems during floods (from 0 to 10) to determine the colours used in the risk level map, as described in Table 3.

**Table 3:** Risk levels and associated map colours

Risk levels	Map colours
10	Dark Red
9	Maroon
8	Red
7	Orange Red
6	Orange
5	Gold
4	Yellow
3	Greenish Yellow
2	Yellowish Green
1	Forest Green
0	Green

**Fig. 4:**
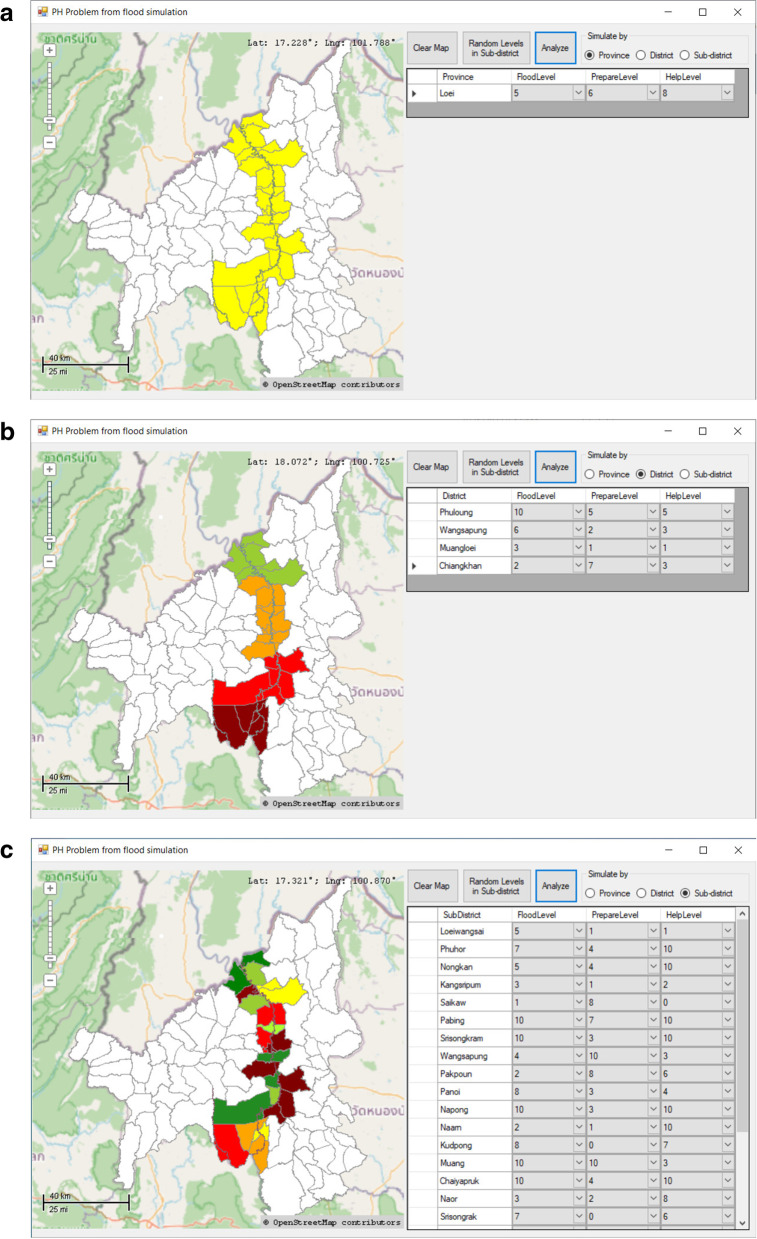
Simulation examples of public health problems during floods

Examples of the public health problem situations simulated by the program developed with Visual Studio 2017 and MapWinGIS version 5.3.0 are shown in Fig. 4 below.

## Discussion

The results indicated that only flood severity had a statistically significant effect on public health problems (*p* < .05), both directly and indirectly, as also reported in several studies [34, 35]. The more disastrous a flood situation becomes, the more serious the public health problems will be. On the other hand, if flood situations are less disastrous, the public health problems are also less serious. During severe floods, many issues can occur, such as food and water scarcity, consumption of contaminated food and water, unsanitary excretion, flooded houses, power outage, poisonous animals in floodwater, insects carrying diseases from floodwater, and communication outages. These issues can lead to public health problems, including malnutrition from food and water scarcity, poisoning and water-borne diseases from consuming contaminated food and water, water-borne diseases due to water contamination from unsanitary excretion, contagious diseases transmitted from poisonous animals and insects in floodwater, drowning because of the high level of floodwater level, injuries from uncontrolled electrical currents, accidents in the dark due to power outages, and mental health problems from a lack of communication with the outside world. Mental health problems encountered during floods include stress, panic, and fear; moreover, mental health problems such as depression persist even after floods. As indicated by the results, mental health problems differed from other problems, as mental health problems were not present during floods in the Loei River Basin. Since the mass of floodwater quickly flowed into the Mekong River, the duration of each flood in the basin usually lasted no more than 2 days; subsequently, mental health was not yet affected by floods.

Help had a direct inverse effect on public health problems, which was supported by previous studies [36, 37]. When there was a great deal of help, the number of public health problems was lower. In contrast, if help was limited, public health problems became more serious. Help could clearly relieve public health problems. For instance, food and water aid can decrease the risks of malnutrition, food and water poisoning, and infections of diseases from food and water because the donated food and water were prepared and brought in from outside the affected area and hence were not contaminated with floodwater. Rescuing and moving people, patients, and their belongings out of the affected area ensured that they would be safe from the source of public health problems. Rescued and transferred patients could also receive the care they needed immediately. Saving victims’ possessions reduces the loss of property, which can also lower the chances of mental health issues. In addition, using public relations to keep those affected informed can help them be aware of possible harms from floods, resulting in fewer public health issues.

Preparation had both direct and indirect inverse impacts on public health problems, as concluded in other studies [38, 39]. Public health problems were less common when there was more preparation. On the other hand, public health problems were more severe when preparation was insufficient. Preparedness could directly reduce public health problems. For instance, if food and water were stored in advance, there would not be a shortage of food and water during a flood. The indirect impact of preparation involved help. If the aid plan were well prepared, rescue would be prompt in case of emergency. According to the results, the direct impact had a minimal value because preparation primarily led to the indirect impact in the form of help. During a flood disaster, good preparedness plays a crucial role in providing sufficient and effective assistance that can reduce public health problems.

Although help and preparation directly and indirectly affected public health problems, they did not have a statistically significant effect. The standardized factor loading is very low, which may indicate that factors other than flood severity, help and preparation could affect the occurrence of public health problems, which is an interesting point for future study. However, the observed variable, which is a component of all latent variables including the public health problem latent variable, flood severity latent variable, preparation latent variable, and help latent variable, was statistically significant (*p* < .05). Therefore, the addition of latent variables from the existing study may enhance the predictive ability and statistical significance of future studies.

In terms of using a developed application to simulate situations of public health problems during floods, there has been a multitude of studies simulating flood situations [26–28, 39]. In this study, GIS and SEM techniques were used to combine values for public health problem simulation. The advantage of SEM over the regression equation is that SEM considers latent variables with observed variables as a factor, whereas regression equations examine only observed variables measured by collecting data. Another advantage of SEM with path analysis is that it calculates not only direct but also indirect effects, resulting in a more elaborate consideration of effects. In contrast, the regression equation examines only the direct effect. Furthermore, the incorporation of GIS with SEM allows the mapping arrangement to be visualized, supporting more convenient and efficient management of public health problems at both the provincial and subdistrict levels. Nonetheless, some issues were not considered in this study, and the predictive ability was only 7.7%, probably due to the high complexity of public health issues. Nonetheless, this research provides a good starting point for further study and development to clarify, manage, and solve public health problems. A more diverse study of related variables could be developed in future research, which would likely increase the model’s predictive ability.

Management to address the three latent variables affecting public health problems—flood severity, preparation and help—could be practically implemented. Water management through river dams and the tributary reservoirs surrounding the province that connect with the Loei River should be considered to reduce flood severity. Preparation in terms of both budget and manpower, including various equipment that supports the provision of emergency assistance, should also be considered. In providing help for flood incidents, budget and manpower must be managed, directed, and facilitated effectively across all sectors, including government agencies, the private sector, and the people, who must work together vigorously with dedication and full efficiency.

The simulation model of public health problems during a flood can be implemented at both the technical and policy levels in different areas of Thailand. Questionnaires can be collected in a given area, and simulations of public health problems in a flood situation within that area can then be projected based on questionnaire data. Following simulation, area situation data can be used for preparation planning, assistance, and fixing flood-related problems that arise in that area.

### Limitations

Since the simulation system for public health problem situations was developed using cross-sectional data, the accuracy of the predictions could not be evaluated due to the lack of data for comparison. Therefore, in future studies, longitudinal data should be consecutively collected for at least 2 years for comparison to examine the prediction accuracy of the simulation system.

## Conclusions

This study found that flood severity and public health problems were positively correlated, but preparation and help had inverse relationships with public health problems. The variance in the public health problems could be predicted by 7.7%. When the standardized factor loadings from the analysis were applied in the system to simulate public health problem situations during floods, GIS was also adopted to simulate situations at the province, district, and subdistrict levels via the simulation application. Nevertheless, since the data were cross-sectional, the prediction accuracy could not be assessed owing to the lack of comparable data. For further study, longitudinal data should be gathered to evaluate the effectiveness of the simulation system.

## Supplementary Information

**Additional file 1.**

**Additional file 2.**

**Additional file 3.**

**Additional file 4.**

**Additional file 5.**

## Data Availability

The datasets used and/or analysis during the current study are available from the corresponding author upon reasonable request.

## Abbreviation

IOC: Index of item-objective congruence
