# Supplementary material for: Application for simulating public health problems during floods around the Loei River in Thailand: the implementation of a geographic information system and structural equation model
Source: BMC Public Health. 2022 Aug 31;22:1651. doi: 10.1186/s12889-022-14018-7 (PMC9429490; doi:10.1186/s12889-022-14018-7)
Supplement: Supplementary file 1 — Additional file 1. [file 12889_2022_14018_MOESM1_ESM.pdf]

ID____________

Questionnaire

Application for Simulating Public Health Problems during Floods around the Loei River in Thailand: The Implementation of a Geographic Information System and Structural Equation Model

---

The objectives of this study are to investigate the causal model among public health problems during floods, flood severity, preparation, and help and to develop an application with structural equation model and geographic information system to simulate public health problems around the Loei River during floods.

The results will be summarized as a whole. Therefore, the answers from this questionnaire will be the great benefit in being a guideline for preventing public health problems caused by flooding as well as beneficial to the general public.

This questionnaire is divided into 5 parts as follows:

Part 1 General information

Part 2 Preparation

Part 3 Help

Part 4 Flood severity

Part 5 Public Health Problem

Thank you for your kind cooperation in answering this questionnaire.

Managed research by

Asst. Prof. Dr. Tanunchai Boonnuk et. al.
Public Health Program
Faculty of Science and Technology, Loei Rajabhat University, Thailand

Questionnaire

District…………..……….…….. Sub District……………..…………….

Part 1 General information

1. Gender ☐1 Male ☐2 Female

2. Age ………. years old

3. Marital status ☐1 Single ☐2 Married ☐3 Widowed/divorced/separated

4. Education ☐1 None ☐2 Elementary ☐3 High school
☐4 Diploma/Bachelor's degree ☐5 Master's degree or higher

5. Occupation ☐1 Unemployed ☐2 Freelancer ☐3 Farmer ☐4 Merchant/vender
☐5 Civil servant ☐6 Others

6. Average monthly income …………………………. Baht

7. Number of household member(s) ……….

Part 2 Preparation

How do the relevant agencies prepare for flooding in your household?

**Preparation**	**Preparation Level**										
	**0**	**1**	**2**	**3**	**4**	**5**	**6**	**7**	**8**	**9**	**10**
1. Food preparation											
2. Water preparation											
3. Moving things preparation											
4. Transport patient preparation											

Part 3 Help

How much does the flooded area in your household received the assistance of the relevant agencies from the flood?

**Help**	**Help Level**										
	**0**	**1**	**2**	**3**	**4**	**5**	**6**	**7**	**8**	**9**	**10**
1. Food and water											
2. Transportation											
3. Public relations											
4. Interpersonal communication											

Part 4 Flood severity

How much are the flooded area in your household affected by the flood?

**Flood Severity**	**Severity Level**										
	**0**	**1**	**2**	**3**	**4**	**5**	**6**	**7**	**8**	**9**	**10**
1. Food scarcity											
2. Water scarcity											
3. Contaminated food consumption											
4. Contaminated water consumption											
5. Unsanitary excretion											
6. Flooded houses											
7. Power outage											
8. Poisonous animals in floodwater											
9. Insects carrying diseases from floodwater											
10. Communication unavailability											

Part 5 Public Health Problem

How many public health problems does your household suffer from floods?

**Public Health Problem**	**Problem Level**										
	**0**	**1**	**2**	**3**	**4**	**5**	**6**	**7**	**8**	**9**	**10**
1. Drowning											
2. Electric current injury											
3. Accident injury											
4. Malnutrition											
5. Poisoning											
6. Contagious											
7. Poisonous animal bites											
8. Stressed											
9. Depressed											
10. Shocked/Fear											

Thank you for your cooperation in answering this questionnaire.
