# Supplementary material for: Application for simulating public health problems during floods around the Loei River in Thailand: the implementation of a geographic information system and structural equation model
Source: BMC Public Health. 2022 Aug 31;22:1651. doi: 10.1186/s12889-022-14018-7 (PMC9429490; doi:10.1186/s12889-022-14018-7)
Supplement: Supplementary file 3 — Additional file 3. [file 12889_2022_14018_MOESM3_ESM.pdf]

```
DATASET ACTIVATE DataSet1.

FREQUENCIES VARIABLES=district subdist gender agegr status edu occupa incgr n
omemgr
  /ORDER=ANALYSIS.
```

Frequencies

[DataSet1] D:\วิดีโอและเอกสารเพิ่มเติมวิจัยน้ำท่วม\flooddata.sav

Statistics

		District	Sub District	Gender	Age Group	Marital Status	Education
N	Valid	560	560	560	560	560	560
	Missing	0	0	0	0	0	0

Statistics

		Occupation	Income Group	Number Of Member Group
N	Valid	560	560	560
	Missing	0	0	0

Frequency Table

District

		Frequency	Percent	Valid Percent	Cumulative Percent
Valid	Chiangkhan	134	23.9	23.9	23.9
	Muangloei	147	26.3	26.3	50.2
	Phuloung	126	22.5	22.5	72.7
	Wangsapung	153	27.3	27.3	100.0
	Total	560	100.0	100.0	

Sub District

		Frequency	Percent	Valid Percent	Cumulative Percent
Valid	Chaiyapruk	22	3.9	3.9	3.9
	Chiangkhan	24	4.3	4.3	8.2
	Hadsaikaw	27	4.8	4.8	13.0
	Jomsri	10	1.8	1.8	14.8
	Kangsripum	38	6.8	6.8	21.6
	Kudpong	13	2.3	2.3	23.9
	Loeiwangsai	68	12.1	12.1	36.1
	Muang	17	3.0	3.0	39.1
	Naarn	15	2.7	2.7	41.8
	Nakam	18	3.2	3.2	45.0
	Naor	19	3.4	3.4	48.4
	Napong	14	2.5	2.5	50.9
	Nasow	30	5.4	5.4	56.3
	Nongkan	9	1.6	1.6	57.9
	Pabing	23	4.1	4.1	62.0
	Pakpoun	21	3.8	3.8	65.7
	Paktom	16	2.9	2.9	68.6
	Panoi	19	3.4	3.4	72.0
	Phuhor	11	2.0	2.0	73.9
	Saikaw	40	7.1	7.1	81.1
	Srisongkram	21	3.8	3.8	84.8
	Srisongrak	29	5.2	5.2	90.0
	Tad	27	4.8	4.8	94.8
	Wangsapung	29	5.2	5.2	100.0
	Total	560	100.0	100.0	

Gender

		Frequency	Percent	Valid Percent	Cumulative Percent
Valid	Male	208	37.1	37.1	37.1
	Female	352	62.9	62.9	100.0
	Total	560	100.0	100.0	

Age Group

		Frequency	Percent	Valid Percent	Cumulative Percent
Valid	Under 35 years old	83	14.8	14.8	14.8
	35-59 years old	258	46.1	46.1	60.9
	60 years old and over	219	39.1	39.1	100.0
	Total	560	100.0	100.0	

Marital Status

		Frequency	Percent	Valid Percent	Cumulative Percent
Valid	Single	70	12.5	12.5	12.5
	Married	477	85.2	85.2	97.7
	Widowed/divorced/separated	13	2.3	2.3	100.0
	Total	560	100.0	100.0	

Education

		Frequency	Percent	Valid Percent	Cumulative Percent
Valid	None	20	3.6	3.6	3.6
	Elementary	398	71.1	71.1	74.6
	High school	113	20.2	20.2	94.8
	Diploma/Bachelor’s degree	28	5.0	5.0	99.8
	Master’s degree or higher	1	.2	.2	100.0
	Total	560	100.0	100.0	

Occupation

		Frequency	Percent	Valid Percent	Cumulative Percent
Valid	Unemployed	100	17.9	17.9	17.9
	Freelancer	68	12.1	12.1	30.0
	Farmer	298	53.2	53.2	83.2
	Merchant/vender	68	12.1	12.1	95.4
	Civil servant	9	1.6	1.6	97.0
	Others	17	3.0	3.0	100.0
	Total	560	100.0	100.0	

Income Group

		Frequency	Percent	Valid Percent	Cumulative Percent
Valid	No income	47	8.4	8.4	8.4
	Less than 1,000 Baht	115	20.5	20.5	28.9
	1,001-10,000 Baht	349	62.3	62.3	91.3
	More than 10,000 Baht	49	8.8	8.8	100.0
	Total	560	100.0	100.0	

Number Of Member Group

		Frequency	Percent	Valid Percent	Cumulative Percent
Valid	1-3 member(s)	118	21.1	21.1	21.1
	4-6 members	360	64.3	64.3	85.4
	7 members or over	82	14.6	14.6	100.0
	Total	560	100.0	100.0	

```
DESCRIPTIVES VARIABLES=age nomem
  /STATISTICS=MEAN STDDEV MIN MAX.
```

Descriptives

[DataSet1] D:\วิดีโอและเอกสารเพิ่มเติมวิจัยน้ำท่วม\flooddata.sav

Descriptive Statistics

	N	Minimum	Maximum	Mean	Std. Deviation
Age	560	20	96	53.23	16.508
Number Of Member	560	1	11	4.79	1.660
Valid N (listwise)	560				

```
FREQUENCIES VARIABLES=income
  /FORMAT=NOTABLE
  /STATISTICS=MINIMUM MAXIMUM MEDIAN
  /ORDER=ANALYSIS.
```

Frequencies

[DataSet1] D:\วิดีโอและเอกสารเพิ่มเติมวิจัยน้ำท่วม\flooddata.sav

Statistics

Income

N	Valid	560
	Missing	0
Median		3000.00
Minimum		0
Maximum		60000
