# Supplementary material for: Application for simulating public health problems during floods around the Loei River in Thailand: the implementation of a geographic information system and structural equation model
Source: BMC Public Health. 2022 Aug 31;22:1651. doi: 10.1186/s12889-022-14018-7 (PMC9429490; doi:10.1186/s12889-022-14018-7)
Supplement: Supplementary file 4 — Additional file 4. [file 12889_2022_14018_MOESM4_ESM.pdf]

Mplus VERSION 7.4
MUTHEN & MUTHEN
03/31/2022   7:59 PM

INPUT INSTRUCTIONS

```
  TITLE:  Flood

  DATA:
    FILE IS "D:\flood.dat";

  VARIABLE:
    NAMES ARE prep1-prep4 help1-help4 fprb1-fprb10 phprb1-phprb10;
    USEVARIABLES ARE prep1-prep4 help1-help4 fprb1-fprb10 phprb1-phprb10;
    !CATEGORICAL b1-b7;

  ANALYSIS:
    TYPE IS GENERAL;
    ESTIMATOR IS ML;
    ITERATIONS = 20000;
    CONVERGENCE = 0.00001;

  MODEL:
    prep BY prep1-prep4;
    help BY help1-help4;
    fprb BY fprb1-fprb10;
    phprb BY phprb1-phprb10;
    help ON prep;
    phprb ON prep fprb help;
    FPRB2    WITH FPRB1;
    FPRB4   WITH    FPRB3;
    PHPRB10 WITH    PHPRB9;
    PHPRB7  WITH    PHPRB6;
    PREP4   WITH    PREP3;
    HELP2   WITH    HELP1;
    PHPRB2  WITH    PHPRB1;
    FPRB9   WITH    FPRB8;
    FPRB10  WITH    FPRB9;
    FPRB    WITH    HELP;
    PHPRB5  WITH    PHPRB4;
    FPRB10  WITH    FPRB8;
    PHPRB6  WITH    PHPRB5;
    PHPRB7  WITH    PHPRB5;
    FPRB7   WITH    FPRB5;
    PREP3   WITH    PREP2;
    PHPRB8  WITH    PHPRB7;
    PHPRB8  WITH    PHPRB6;
    PHPRB6  WITH    FPRB9;
    PHPRB7  WITH    FPRB9;
    PHPRB6  WITH    PHPRB4;
    PHPRB7  WITH    PHPRB4;
    PHPRB8  WITH    FPRB6;
    PHPRB8  WITH    PHPRB5;
    PHPRB9  WITH    FPRB8;
    PHPRB9  WITH    PHPRB8;
    PHPRB10 WITH    PHPRB8;
    PHPRB8  WITH    FPRB8;
    HELP1   WITH    PREP1;
    HELP2   WITH    PREP1;
    PHPRB3  WITH    PHPRB2;
    PHPRB10 WITH    FPRB8;
    PHPRB4  WITH    PREP3;
    PHPRB4  WITH    PREP4;
    FPRB8   WITH    FPRB5;
```

```
    PHPRB2  WITH    FPRB2;
    PHPRB2  WITH    FPRB9;
    PHPRB2  WITH    FPRB7;
    PHPRB6  WITH    FPRB2;
    PHPRB5  WITH    FPRB3;
    PHPRB5  WITH    FPRB9;
    PHPRB5  WITH    FPRB4;
    FPRB5   WITH    FPRB4;
    FPRB5   WITH    HELP3;
    HELP2   WITH    PREP2;
    HELP1   WITH    PREP2;
    PREP2   WITH    PREP1;
    PHPRB4  WITH    HELP3;
    PHPRB6  WITH    FPRB10;
    PHPRB5  WITH    FPRB10;
    PHPRB7  WITH    FPRB10;
    PHPRB4  WITH    FPRB8;
    PHPRB8  WITH    FPRB10;
    PHPRB8  WITH    PREP4;
    FPRB2   WITH    HELP1;
    PHPRB1  WITH    PREP1;
    PHPRB4  WITH    FPRB10;
    PHPRB9  WITH    PREP3;
    FPRB10  WITH    HELP3;
    FPRB10  WITH    FPRB3;
    FPRB9   WITH    FPRB5;
    FPRB5   WITH    FPRB3;
    PHPRB7  WITH    FPRB5;
    PHPRB7  WITH    FPRB8;
    PHPRB6  WITH    FPRB8;
    PHPRB5  WITH    FPRB8;
    PHPRB4  WITH    FPRB9;
    HELP4   WITH    HELP2;
    PHPRB2  WITH    PREP4;
    PHPRB8  WITH    HELP2;
    PHPRB9  WITH    PHPRB7;
    PHPRB10 WITH    PHPRB7;
    PHPRB7  WITH    PHPRB1;
    PHPRB9  WITH    PHPRB6;
    PHPRB10 WITH    PHPRB6;
    PHPRB5  WITH    FPRB5;
    PHPRB5  WITH    FPRB6;
    PHPRB5  WITH    FPRB7;
    PHPRB10 WITH    FPRB5;
    PHPRB10 WITH    PHPRB3;
    PHPRB10 WITH    HELP4;
    PHPRB9  WITH    FPRB6;
    PHPRB10 WITH    FPRB6;
    FPRB10  WITH    FPRB5;
    FPRB10  WITH    FPRB4;
    FPRB8   WITH    FPRB4;
    FPRB8   WITH    PREP3;
    FPRB5   WITH    PREP3;
    FPRB5   WITH    PREP4;
    PHPRB3  WITH    FPRB6;
    PHPRB3  WITH    FPRB7;
    PHPRB3  WITH    FPRB5;
    PHPRB3  WITH    PREP2;
    PHPRB3  WITH    PREP1;
    PHPRB4  WITH    PHPRB3;
    FPRB10  WITH    FPRB2;
    FPRB7   WITH    FPRB2;
    FPRB7   WITH    FPRB1;
    FPRB4   WITH    PREP3;
```

```
    FPRB9   WITH    PREP2;
    FPRB9   WITH    PREP1;
    PHPRB9  WITH    FPRB9;
    PHPRB10 WITH    FPRB9;
    PHPRB8  WITH    PREP1;
    PHPRB2  WITH    HELP2;
    HELP3   WITH    HELP1;
    FPRB7   WITH    PREP2;
    FPRB6   WITH    PREP2;
    FPRB6 WITH  PREP1;
    FPRB7   WITH    PREP1;
    PHPRB1  WITH    FPRB7;
    PHPRB1  WITH    PREP2;
    PHPRB2  WITH    PREP2;
    PHPRB6  WITH    PREP4;
    PHPRB7  WITH    PREP4;
    PHPRB6  WITH    PREP3;
    PHPRB7  WITH    PREP3;
    PHPRB5  WITH    PREP2;
    PHPRB5  WITH    PREP1;
    PHPRB6  WITH    FPRB5;
    FPRB10  WITH    FPRB7;
    FPRB10  WITH    FPRB6;
    FPRB7   WITH    HELP3;
    PHPRB10 WITH    PHPRB2;
    FPRB6   WITH    HELP3;
    PHPRB4  WITH    HELP2;
    PHPRB4  WITH    PHPRB2;
    PHPRB4  WITH    PHPRB1;
    PHPRB4  WITH    FPRB7;
    PHPRB4  WITH    FPRB6;
    PHPRB6  WITH    FPRB1;
    PHPRB5  WITH    FPRB1;
    PHPRB5  WITH    FPRB2;
    FPRB10  WITH    FPRB1;
    PHPRB6  WITH    FPRB6;
    PHPRB6  WITH    FPRB7;
    PHPRB7  WITH    FPRB7;
    FPRB9   WITH    FPRB7;
    FPRB8   WITH    FPRB7;
    PHPRB8  WITH    FPRB7;
    PHPRB8  WITH    PREP2;
    PHPRB10 WITH    PREP3;
    PHPRB6  WITH    HELP4;
    PHPRB5  WITH    HELP4;
    PHPRB7  WITH    HELP4;
    FPRB9   WITH    HELP4;
    FPRB8   WITH    HELP4;
    FPRB10  WITH    HELP4;
    PHPRB1  WITH    HELP3;
    FPRB8   WITH    HELP3;
    FPRB9   WITH    HELP3;
    PHPRB6  WITH    HELP3;
    PHPRB7  WITH    HELP3;
    PHPRB5  WITH    HELP3;
    PHPRB4  WITH    HELP4;
    FPRB6   WITH    HELP4;
    FPRB6   WITH    PREP3;
    FPRB7   WITH    PREP4;
    FPRB7   WITH    PREP3;
    PHPRB2  WITH    FPRB6;
    PHPRB2  WITH    PREP3;
    FPRB7   WITH    FPRB6;
    PHPRB1  WITH    FPRB5;
```

```
    FPRB8   WITH    FPRB6;
    FPRB9   WITH    FPRB6;
    FPRB6   WITH    FPRB5;
    PHPRB7  WITH    FPRB6;
    PHPRB9  WITH    FPRB5;
    PHPRB8  WITH    FPRB5;
    FPRB7   WITH    HELP4;
    FPRB5   WITH    HELP4;
    PHPRB4  WITH    FPRB2;
    PHPRB3  WITH    FPRB2;
    PHPRB10 WITH    FPRB2;
    PHPRB10 WITH    FPRB1;
    PHPRB3  WITH    FPRB1;
    PHPRB2  WITH    FPRB1;
    PHPRB4  WITH    FPRB1;
    PHPRB6  WITH    PHPRB3;
    FPRB10  WITH    PREP1;
    FPRB8   WITH    PREP1;
    FPRB8   WITH    PREP2;
    FPRB10  WITH    PREP2;
    FPRB8   WITH    PREP4;
    FPRB5   WITH    PREP1;

  OUTPUT:  SAMPSTAT MODINDICES(0) STANDARDIZED;



INPUT READING TERMINATED NORMALLY



Flood

SUMMARY OF ANALYSIS

Number of groups                                                 1
Number of observations                                         560

Number of dependent variables                                   28
Number of independent variables                                  0
Number of continuous latent variables                            4

Observed dependent variables

  Continuous
   PREP1       PREP2       PREP3       PREP4       HELP1       HELP2
   HELP3       HELP4       FPRB1       FPRB2       FPRB3       FPRB4
   FPRB5       FPRB6       FPRB7       FPRB8       FPRB9       FPRB10
   PHPRB1      PHPRB2      PHPRB3      PHPRB4      PHPRB5      PHPRB6
   PHPRB7      PHPRB8      PHPRB9      PHPRB10

Continuous latent variables
   PREP        HELP        FPRB        PHPRB


Estimator                                                       ML
Information matrix                                        OBSERVED
Maximum number of iterations                                 20000
Convergence criterion                                    0.100D-04
Maximum number of steepest descent iterations                   20

Input data file(s)
  D:\flood.dat
```

Input data format FREE

SAMPLE STATISTICS

SAMPLE STATISTICS

Means

	PREP1	PREP2	PREP3	PREP4	HELP1
1	2.721	2.771	3.064	3.013	3.727

Means

	HELP2	HELP3	HELP4	FPRB1	FPRB2
1	3.752	4.043	4.116	2.159	2.180

Means

	FPRB3	FPRB4	FPRB5	FPRB6	FPRB7
1	1.496	1.466	2.677	3.577	2.973

Means

	FPRB8	FPRB9	FPRB10	PHPRB1	PHPRB2
1	3.375	3.159	2.870	0.446	0.445

Means

	PHPRB3	PHPRB4	PHPRB5	PHPRB6	PHPRB7
1	0.561	0.852	1.436	2.948	3.239

Means

	PHPRB8	PHPRB9	PHPRB10
1	5.775	5.714	5.911

Covariances

	PREP1	PREP2	PREP3	PREP4	HELP1
PREP1	4.540				
PREP2	4.435	4.544			
PREP3	4.646	4.752	5.946		
PREP4	4.695	4.721	5.781	6.062	
HELP1	2.529	2.438	2.419	2.471	6.731
HELP2	2.547	2.486	2.464	2.525	6.552
HELP3	2.630	2.642	3.022	3.083	6.156
HELP4	2.706	2.705	3.091	3.154	6.169
FPRB1	-0.158	-0.167	-0.201	-0.239	1.890
FPRB2	-0.189	-0.194	-0.217	-0.254	1.847
FPRB3	-0.255	-0.262	-0.159	-0.137	0.448
FPRB4	-0.179	-0.183	-0.050	-0.050	0.474
FPRB5	0.376	0.446	0.901	0.833	1.997
FPRB6	1.262	1.341	1.433	1.364	3.243
FPRB7	0.601	0.681	0.616	0.588	3.734
FPRB8	0.378	0.468	1.085	1.020	1.847

FPRB9	0.925	1.008	1.679	1.727	2.679
FPRB10	0.546	0.633	1.091	1.116	2.132
PHPRB1	-0.122	-0.114	-0.136	-0.118	-0.060
PHPRB2	-0.140	-0.113	-0.098	-0.061	-0.155
PHPRB3	-0.147	-0.113	-0.129	-0.100	-0.156
PHPRB4	-0.075	-0.071	-0.201	-0.129	0.583
PHPRB5	0.193	0.207	0.636	0.689	0.808
PHPRB6	1.689	1.752	2.685	2.795	1.966
PHPRB7	2.111	2.162	3.163	3.261	2.010
PHPRB8	1.577	1.622	2.436	2.203	1.051
PHPRB9	2.103	2.124	3.049	2.891	1.363
PHPRB10	1.832	1.849	2.665	2.564	1.588

Covariances

	HELP2	HELP3	HELP4	FPRB1	FPRB2
HELP2	6.601				
HELP3	6.159	7.095			
HELP4	6.138	6.813	7.238		
FPRB1	1.913	1.745	1.796	4.130	
FPRB2	1.895	1.755	1.809	4.046	4.176
FPRB3	0.504	0.604	0.664	1.969	2.066
FPRB4	0.530	0.641	0.701	1.997	2.102
FPRB5	2.020	2.139	2.373	3.060	3.107
FPRB6	3.290	3.320	3.463	3.753	3.819
FPRB7	3.772	3.821	4.012	4.340	4.351
FPRB8	1.861	2.513	2.606	2.578	2.582
FPRB9	2.709	3.345	3.474	2.810	2.830
FPRB10	2.166	2.550	2.744	2.835	2.818
PHPRB1	-0.020	-0.069	-0.011	0.272	0.271
PHPRB2	-0.124	-0.130	-0.082	0.226	0.193
PHPRB3	-0.104	-0.088	-0.062	0.338	0.322
PHPRB4	0.647	0.567	0.723	1.102	1.077
PHPRB5	0.853	1.146	1.290	1.397	1.362
PHPRB6	2.041	2.633	2.835	1.540	1.591
PHPRB7	2.063	2.733	2.897	1.205	1.193
PHPRB8	1.042	1.517	1.601	0.680	0.723
PHPRB9	1.411	1.887	1.958	0.740	0.777
PHPRB10	1.626	2.059	2.060	1.000	1.061

Covariances

	FPRB3	FPRB4	FPRB5	FPRB6	FPRB7
FPRB3	3.214				
FPRB4	3.131	3.363			
FPRB5	2.432	2.540	6.983		
FPRB6	2.410	2.445	5.315	9.523	
FPRB7	2.570	2.596	5.179	7.628	11.097
FPRB8	1.555	1.507	4.660	4.862	5.917
FPRB9	1.703	1.710	4.619	5.201	6.395
FPRB10	2.009	1.964	4.335	5.068	6.168
PHPRB1	0.418	0.451	0.509	0.509	0.471
PHPRB2	0.420	0.445	0.472	0.415	0.289
PHPRB3	0.491	0.523	0.472	0.353	0.454
PHPRB4	0.916	0.949	1.284	1.423	1.762
PHPRB5	1.196	1.158	2.077	2.134	2.506
PHPRB6	0.451	0.508	2.446	2.792	3.222
PHPRB7	-0.003	0.042	2.349	2.355	2.753
PHPRB8	-0.299	-0.240	1.733	2.407	1.362
PHPRB9	-0.117	-0.061	1.958	2.877	1.739
PHPRB10	-0.232	-0.182	1.855	3.064	1.980

Covariances

	FPRB8	FPRB9	FPRB10	PHPRB1	PHPRB2
FPRB8	7.327				
FPRB9	6.217	7.348			
FPRB10	5.279	6.110	6.749		
PHPRB1	0.277	0.236	0.255	1.290	
PHPRB2	0.298	0.181	0.263	1.276	1.493
PHPRB3	0.347	0.341	0.389	1.055	1.161
PHPRB4	0.965	1.118	1.300	0.979	1.007
PHPRB5	2.210	2.493	2.573	0.698	0.778
PHPRB6	3.319	4.210	3.422	0.537	0.625
PHPRB7	3.433	3.994	3.072	0.284	0.415
PHPRB8	2.422	1.964	1.483	-0.221	-0.264
PHPRB9	2.670	2.440	2.072	-0.212	-0.235
PHPRB10	2.433	2.532	2.156	-0.215	-0.282

Covariances

	PHPRB3	PHPRB4	PHPRB5	PHPRB6	PHPRB7
PHPRB3	1.457				
PHPRB4	1.253	2.301			
PHPRB5	0.986	1.766	3.653		
PHPRB6	0.700	1.339	3.081	6.428	
PHPRB7	0.412	0.921	2.590	6.073	7.689
PHPRB8	-0.551	-0.589	0.418	2.956	4.032
PHPRB9	-0.527	-0.537	0.671	3.373	4.295
PHPRB10	-0.586	-0.486	0.651	3.447	4.291

Covariances

	PHPRB8	PHPRB9	PHPRB10
PHPRB8	8.778		
PHPRB9	7.739	8.497	
PHPRB10	7.685	7.846	8.831

Correlations

	PREP1	PREP2	PREP3	PREP4	HELP1
PREP1	1.000				
PREP2	0.976	1.000			
PREP3	0.894	0.914	1.000		
PREP4	0.895	0.899	0.963	1.000	
HELP1	0.458	0.441	0.382	0.387	1.000
HELP2	0.465	0.454	0.393	0.399	0.983
HELP3	0.463	0.465	0.465	0.470	0.891
HELP4	0.472	0.472	0.471	0.476	0.884
FPRB1	-0.036	-0.039	-0.041	-0.048	0.358
FPRB2	-0.043	-0.045	-0.044	-0.050	0.348
FPRB3	-0.067	-0.068	-0.036	-0.031	0.096
FPRB4	-0.046	-0.047	-0.011	-0.011	0.100
FPRB5	0.067	0.079	0.140	0.128	0.291
FPRB6	0.192	0.204	0.190	0.180	0.405
FPRB7	0.085	0.096	0.076	0.072	0.432
FPRB8	0.065	0.081	0.164	0.153	0.263
FPRB9	0.160	0.174	0.254	0.259	0.381
FPRB10	0.099	0.114	0.172	0.174	0.316
PHPRB1	-0.050	-0.047	-0.049	-0.042	-0.020
PHPRB2	-0.054	-0.043	-0.033	-0.020	-0.049
PHPRB3	-0.057	-0.044	-0.044	-0.034	-0.050

PHPRB4	-0.023	-0.022	-0.054	-0.034	0.148
PHPRB5	0.047	0.051	0.137	0.146	0.163
PHPRB6	0.313	0.324	0.434	0.448	0.299
PHPRB7	0.357	0.366	0.468	0.478	0.279
PHPRB8	0.250	0.257	0.337	0.302	0.137
PHPRB9	0.339	0.342	0.429	0.403	0.180
PHPRB10	0.289	0.292	0.368	0.350	0.206

Correlations

	HELP2	HELP3	HELP4	FPRB1	FPRB2
HELP2	1.000				
HELP3	0.900	1.000			
HELP4	0.888	0.951	1.000		
FPRB1	0.366	0.322	0.328	1.000	
FPRB2	0.361	0.322	0.329	0.974	1.000
FPRB3	0.109	0.126	0.138	0.540	0.564
FPRB4	0.112	0.131	0.142	0.536	0.561
FPRB5	0.297	0.304	0.334	0.570	0.575
FPRB6	0.415	0.404	0.417	0.598	0.606
FPRB7	0.441	0.431	0.448	0.641	0.639
FPRB8	0.268	0.348	0.358	0.469	0.467
FPRB9	0.389	0.463	0.476	0.510	0.511
FPRB10	0.324	0.369	0.393	0.537	0.531
PHPRB1	-0.007	-0.023	-0.004	0.118	0.117
PHPRB2	-0.039	-0.040	-0.025	0.091	0.077
PHPRB3	-0.033	-0.027	-0.019	0.138	0.131
PHPRB4	0.166	0.140	0.177	0.357	0.347
PHPRB5	0.174	0.225	0.251	0.360	0.349
PHPRB6	0.313	0.390	0.416	0.299	0.307
PHPRB7	0.290	0.370	0.388	0.214	0.210
PHPRB8	0.137	0.192	0.201	0.113	0.119
PHPRB9	0.188	0.243	0.250	0.125	0.130
PHPRB10	0.213	0.260	0.258	0.166	0.175

Correlations

	FPRB3	FPRB4	FPRB5	FPRB6	FPRB7
FPRB3	1.000				
FPRB4	0.952	1.000			
FPRB5	0.513	0.524	1.000		
FPRB6	0.436	0.432	0.652	1.000	
FPRB7	0.430	0.425	0.588	0.742	1.000
FPRB8	0.320	0.304	0.652	0.582	0.656
FPRB9	0.350	0.344	0.645	0.622	0.708
FPRB10	0.431	0.412	0.631	0.632	0.713
PHPRB1	0.205	0.216	0.169	0.145	0.124
PHPRB2	0.192	0.198	0.146	0.110	0.071
PHPRB3	0.227	0.236	0.148	0.095	0.113
PHPRB4	0.337	0.341	0.320	0.304	0.349
PHPRB5	0.349	0.330	0.411	0.362	0.394
PHPRB6	0.099	0.109	0.365	0.357	0.381
PHPRB7	-0.001	0.008	0.321	0.275	0.298
PHPRB8	-0.056	-0.044	0.221	0.263	0.138
PHPRB9	-0.022	-0.012	0.254	0.320	0.179
PHPRB10	-0.044	-0.033	0.236	0.334	0.200

Correlations

	FPRB8	FPRB9	FPRB10	PHPRB1	PHPRB2
FPRB8	1.000				

FPRB9	0.847	1.000			
FPRB10	0.751	0.868	1.000		
PHPRB1	0.090	0.077	0.086	1.000	
PHPRB2	0.090	0.055	0.083	0.920	1.000
PHPRB3	0.106	0.104	0.124	0.770	0.787
PHPRB4	0.235	0.272	0.330	0.568	0.543
PHPRB5	0.427	0.481	0.518	0.322	0.333
PHPRB6	0.484	0.613	0.520	0.187	0.202
PHPRB7	0.457	0.531	0.426	0.090	0.122
PHPRB8	0.302	0.245	0.193	-0.066	-0.073
PHPRB9	0.338	0.309	0.274	-0.064	-0.066
PHPRB10	0.303	0.314	0.279	-0.064	-0.078

Correlations

	PHPRB3	PHPRB4	PHPRB5	PHPRB6	PHPRB7
PHPRB3	1.000				
PHPRB4	0.684	1.000			
PHPRB5	0.427	0.609	1.000		
PHPRB6	0.229	0.348	0.636	1.000	
PHPRB7	0.123	0.219	0.489	0.864	1.000
PHPRB8	-0.154	-0.131	0.074	0.394	0.491
PHPRB9	-0.150	-0.121	0.120	0.456	0.531
PHPRB10	-0.163	-0.108	0.115	0.458	0.521

Correlations

	PHPRB8	PHPRB9	PHPRB10
PHPRB8	1.000		
PHPRB9	0.896	1.000	
PHPRB10	0.873	0.906	1.000

UNIVARIATE SAMPLE STATISTICS

UNIVARIATE HIGHER-ORDER MOMENT DESCRIPTIVE STATISTICS

Variable/ Sample Size	Mean/ Variance	Skewness/ Kurtosis	Minimum/ Maximum	% with Min/Max	Percentiles 20%/60%	40%/80%	Median
PREP1	2.721	1.063	0.000	10.71%	1.000	2.000	2.000
560.000	4.540	0.933	10.000	0.89%	3.000	4.000	
PREP2	2.771	1.026	0.000	10.89%	1.000	2.000	2.000
560.000	4.544	0.874	10.000	0.89%	3.000	4.000	
PREP3	3.064	0.751	0.000	13.21%	1.000	2.000	2.000
560.000	5.946	-0.227	10.000	0.89%	3.000	5.000	
PREP4	3.013	0.763	0.000	15.00%	1.000	2.000	2.000
560.000	6.062	-0.198	10.000	1.07%	3.000	5.000	
HELP1	3.727	0.513	0.000	8.75%	1.000	3.000	3.000
560.000	6.731	-0.549	10.000	2.32%	4.000	6.000	
HELP2	3.752	0.510	0.000	8.75%	1.000	3.000	3.000
560.000	6.601	-0.510	10.000	2.32%	4.000	6.000	
HELP3	4.043	0.377	0.000	8.04%	1.000	3.000	

```
    4.000
             560.000        7.095       -0.705       10.000     2.86%         5.000        6.000
     HELP4                  4.116        0.336        0.000     8.21%         1.000        3.000
    4.000
             560.000        7.238       -0.754       10.000     3.04%         5.000        7.000
     FPRB1                  2.159        1.244        0.000    24.64%         0.000        1.000
    2.000
             560.000        4.130        1.644       10.000     0.36%         2.000        3.000
     FPRB2                  2.180        1.242        0.000    24.46%         0.000        1.000
    2.000
             560.000        4.176        1.619       10.000     0.36%         2.000        3.000
     FPRB3                  1.496        1.413        0.000    42.50%         0.000        0.000
    1.000
             560.000        3.214        1.999        9.000     0.36%         2.000        3.000
     FPRB4                  1.466        1.508        0.000    43.93%         0.000        0.000
    1.000
             560.000        3.363        2.187        9.000     0.36%         1.000        3.000
     FPRB5                  2.677        1.167        0.000    22.32%         0.000        1.000
    2.000
             560.000        6.983        0.644       10.000     3.04%         3.000        5.000
     FPRB6                  3.577        0.847        0.000    13.39%         1.000        2.000
    3.000
             560.000        9.523       -0.404       10.000     9.64%         3.000        6.000
     FPRB7                  2.973        1.019        0.000    33.57%         0.000        1.000
    2.000
             560.000       11.097       -0.226       10.000    10.54%         3.000        6.000
     FPRB8                  3.375        0.645        0.000    16.79%         1.000        2.000
    3.000
             560.000        7.327       -0.425       10.000     2.50%         4.000        6.000
     FPRB9                  3.159        0.839        0.000    16.61%         1.000        2.000
    3.000
             560.000        7.348       -0.163       10.000     2.68%         3.000        5.000
     FPRB10                 2.870        0.900        0.000    19.29%         1.000        2.000
    2.000
             560.000        6.749       -0.012       10.000     1.79%         3.000        5.000
     PHPRB1                 0.446        4.257        0.000    77.32%         0.000        0.000
    0.000
             560.000        1.290       23.474        9.000     0.54%         0.000        1.000
     PHPRB2                 0.445        4.629        0.000    79.11%         0.000        0.000
    0.000
             560.000        1.493       26.982       10.000     0.36%         0.000        1.000
     PHPRB3                 0.561        3.169        0.000    73.04%         0.000        0.000
    0.000
             560.000        1.457       13.085        9.000     0.36%         0.000        1.000
     PHPRB4                 0.852        2.118        0.000    66.79%         0.000        0.000
    0.000
             560.000        2.301        4.861        9.000     0.36%         0.000        2.000
     PHPRB5                 1.436        1.349        0.000    51.25%         0.000        0.000
    0.000
             560.000        3.653        1.302        9.000     0.54%         1.000        3.000
     PHPRB6                 2.948        0.411        0.000    27.32%         0.000        2.000
    3.000
             560.000        6.428       -0.894       10.000     0.18%         4.000        5.000
     PHPRB7                 3.239        0.521        0.000    24.64%         0.000        2.000
    3.000
             560.000        7.689       -0.723       10.000     1.61%         4.000        6.000
     PHPRB8                 5.775       -0.084        0.000     3.04%         3.000        5.000
    6.000
             560.000        8.778       -1.166       10.000    14.82%         7.000        9.000
     PHPRB9                 5.714       -0.026        0.000     2.86%         3.000        4.000
    6.000
             560.000        8.497       -1.127       10.000    14.46%         7.000        9.000
     PHPRB10                5.911       -0.128        0.000     2.86%         3.000        5.000
    6.000
```

```
            560.000       8.831      -1.175      10.000   16.61%       7.000       9.000


THE MODEL ESTIMATION TERMINATED NORMALLY

     WARNING:  THE RESIDUAL COVARIANCE MATRIX (THETA) IS NOT POSITIVE DEFINITE.
     THIS COULD INDICATE A NEGATIVE VARIANCE/RESIDUAL VARIANCE FOR AN OBSERVED
     VARIABLE, A CORRELATION GREATER OR EQUAL TO ONE BETWEEN TWO OBSERVED
     VARIABLES, OR A LINEAR DEPENDENCY AMONG MORE THAN TWO OBSERVED VARIABLES.
     CHECK THE RESULTS SECTION FOR MORE INFORMATION.
     PROBLEM INVOLVING VARIABLE FPRB7.




MODEL FIT INFORMATION

Number of Free Parameters                      274

Loglikelihood

          H0 Value                      -24114.750
          H1 Value                      -23874.871

Information Criteria

          Akaike (AIC)                   48777.500
          Bayesian (BIC)                 49963.355
          Sample-Size Adjusted BIC       49093.545
            (n* = (n + 2) / 24)

Chi-Square Test of Model Fit

          Value                            479.757
          Degrees of Freedom                   160
          P-Value                           0.0000

RMSEA (Root Mean Square Error Of Approximation)

          Estimate                           0.060
          90 Percent C.I.                    0.054  0.066
          Probability RMSEA <= .05           0.005

CFI/TLI

          CFI                                0.985
          TLI                                0.966

Chi-Square Test of Model Fit for the Baseline Model

          Value                          22297.332
          Degrees of Freedom                   378
          P-Value                           0.0000

SRMR (Standardized Root Mean Square Residual)

          Value                              0.127



MODEL RESULTS

                                                  Two-Tailed
                    Estimate       S.E.  Est./S.E.    P-Value
```

```
 PREP     BY
    PREP1              1.000      0.000    999.000    999.000
    PREP2              0.997      0.010     97.649      0.000
    PREP3              1.072      0.039     27.263      0.000
    PREP4              1.100      0.040     27.493      0.000

 HELP     BY
    HELP1              1.000      0.000    999.000    999.000
    HELP2              1.020      0.013     76.332      0.000
    HELP3              1.084      0.023     47.052      0.000
    HELP4              1.090      0.027     40.734      0.000

 FPRB     BY
    FPRB1              1.000      0.000    999.000    999.000
    FPRB2              1.020      0.014     73.570      0.000
    FPRB3              0.675      0.050     13.491      0.000
    FPRB4              0.684      0.051     13.391      0.000
    FPRB5              0.981      0.076     12.965      0.000
    FPRB6              1.217      0.090     13.498      0.000
    FPRB7              1.374      0.101     13.540      0.000
    FPRB8              0.759      0.070     10.809      0.000
    FPRB9              0.826      0.069     12.011      0.000
    FPRB10             0.719      0.090      8.023      0.000

 PHPRB    BY
    PHPRB1             1.000      0.000    999.000    999.000
    PHPRB2             1.109      0.052     21.281      0.000
    PHPRB3             1.454      0.116     12.584      0.000
    PHPRB4             3.066      0.410      7.484      0.000
    PHPRB5             0.937      0.085     11.005      0.000
    PHPRB6             0.844      0.104      8.133      0.000
    PHPRB7             0.517      0.107      4.835      0.000
    PHPRB8            -0.390      0.104     -3.760      0.000
    PHPRB9            -0.378      0.098     -3.854      0.000
    PHPRB10           -0.351      0.096     -3.678      0.000

 HELP     ON
    PREP               0.512      0.046     11.116      0.000

 PHPRB    ON
    PREP              -0.003      0.011     -0.303      0.762
    FPRB               0.145      0.027      5.367      0.000
    HELP              -0.010      0.012     -0.888      0.375

 FPRB     WITH
    HELP               1.545      0.176      8.792      0.000
    PREP              -0.055      0.157     -0.350      0.726

 FPRB2    WITH
    FPRB1              1.038      0.178      5.828      0.000
    HELP1             -0.023      0.009     -2.565      0.010

 FPRB4    WITH
    FPRB3              1.828      0.132     13.849      0.000
    PREP3              0.021      0.013      1.564      0.118

 PHPRB10  WITH
    PHPRB9             7.603      0.468     16.231      0.000
    PHPRB8             7.760      0.491     15.789      0.000
    FPRB8              0.681      0.208      3.284      0.001
    PHPRB7             3.085      0.307     10.054      0.000
    PHPRB6             2.054      0.228      8.999      0.000
    FPRB5              0.670      0.239      2.800      0.005
```

```
   PHPRB3            -0.082      0.034     -2.425      0.015
   HELP4             -0.060      0.040     -1.517      0.129
   FPRB6              1.585      0.262      6.052      0.000
   FPRB9              0.289      0.104      2.772      0.006
   PHPRB2            -0.034      0.023     -1.498      0.134
   PREP3              0.251      0.066      3.824      0.000
   FPRB2              0.241      0.077      3.140      0.002
   FPRB1              0.216      0.077      2.814      0.005

 PHPRB7   WITH
   PHPRB6             4.611      0.260     17.703      0.000
   PHPRB5             1.899      0.182     10.419      0.000
   FPRB9              2.515      0.240     10.468      0.000
   PHPRB4            -0.285      0.236     -1.209      0.227
   FPRB10             1.707      0.224      7.632      0.000
   FPRB5              1.139      0.201      5.661      0.000
   FPRB8              2.166      0.240      9.015      0.000
   PHPRB1            -0.079      0.026     -2.987      0.003
   PREP4              0.677      0.097      7.006      0.000
   PREP3              0.676      0.091      7.403      0.000
   FPRB7              1.171      0.263      4.453      0.000
   HELP4              0.639      0.117      5.436      0.000
   HELP3              0.502      0.111      4.533      0.000
   FPRB6              0.618      0.241      2.568      0.010

 PREP4    WITH
   PREP3              0.412      0.158      2.610      0.009

 HELP2    WITH
   HELP1              1.001      0.074     13.464      0.000
   PREP1              0.389      0.045      8.600      0.000
   PREP2              0.329      0.043      7.727      0.000

 PHPRB2   WITH
   PHPRB1             0.469      0.062      7.514      0.000
   FPRB2             -0.105      0.034     -3.046      0.002
   FPRB9             -0.073      0.019     -3.797      0.000
   FPRB7             -0.223      0.098     -2.268      0.023
   PREP4              0.068      0.031      2.183      0.029
   HELP2             -0.014      0.009     -1.573      0.116
   PREP2              0.016      0.019      0.860      0.390
   FPRB6             -0.124      0.047     -2.651      0.008
   PREP3              0.050      0.031      1.622      0.105
   FPRB1             -0.071      0.034     -2.053      0.040

 FPRB9    WITH
   FPRB8              3.958      0.303     13.082      0.000
   FPRB5              1.714      0.222      7.704      0.000
   PREP2             -0.439      0.080     -5.472      0.000
   PREP1             -0.509      0.083     -6.103      0.000
   FPRB7              2.747      0.336      8.180      0.000
   HELP4              0.750      0.117      6.429      0.000
   HELP3              0.645      0.110      5.854      0.000
   FPRB6              1.462      0.275      5.316      0.000

 FPRB10   WITH
   FPRB9              3.975      0.310     12.801      0.000
   FPRB8              3.275      0.289     11.346      0.000
   HELP3              0.431      0.104      4.130      0.000
   FPRB3              0.515      0.135      3.812      0.000
   FPRB5              1.831      0.273      6.699      0.000
   FPRB4              0.469      0.138      3.409      0.001
   FPRB2              0.397      0.198      2.006      0.045
   FPRB7              3.065      0.393      7.792      0.000
```

```
   FPRB6              1.860      0.341      5.458      0.000
   FPRB1              0.443      0.196      2.261      0.024
   HELP4              0.603      0.112      5.385      0.000
   PREP1             -0.374      0.079     -4.714      0.000
   PREP2             -0.288      0.076     -3.790      0.000

 PHPRB5   WITH
   PHPRB4            -0.301      0.247     -1.216      0.224
   FPRB3              0.767      0.099      7.761      0.000
   FPRB9              1.997      0.181     11.035      0.000
   FPRB4              0.708      0.101      7.035      0.000
   FPRB10             2.102      0.181     11.613      0.000
   FPRB8              1.655      0.179      9.266      0.000
   FPRB5              1.331      0.162      8.236      0.000
   FPRB6              1.200      0.200      6.003      0.000
   FPRB7              1.785      0.223      8.016      0.000
   PREP2             -0.385      0.059     -6.493      0.000
   PREP1             -0.382      0.061     -6.306      0.000
   FPRB1              0.732      0.121      6.070      0.000
   FPRB2              0.691      0.120      5.781      0.000
   HELP4              0.490      0.086      5.698      0.000
   HELP3              0.357      0.081      4.393      0.000

 PHPRB6   WITH
   PHPRB5             2.221      0.173     12.817      0.000
   FPRB9              2.834      0.225     12.616      0.000
   PHPRB4            -0.560      0.286     -1.957      0.050
   FPRB2              0.463      0.091      5.113      0.000
   FPRB10             2.157      0.210     10.286      0.000
   FPRB8              2.059      0.215      9.596      0.000
   PREP4              0.745      0.087      8.516      0.000
   PREP3              0.701      0.082      8.531      0.000
   FPRB5              1.112      0.175      6.364      0.000
   FPRB1              0.407      0.090      4.514      0.000
   FPRB6              1.014      0.219      4.621      0.000
   FPRB7              1.622      0.253      6.415      0.000
   HELP4              0.713      0.109      6.526      0.000
   HELP3              0.527      0.103      5.118      0.000
   PHPRB3            -0.069      0.048     -1.445      0.148

 FPRB7    WITH
   FPRB5              0.929      0.277      3.351      0.001
   FPRB2             -0.012      0.203     -0.059      0.953
   FPRB1              0.039      0.200      0.194      0.846
   PREP2             -0.400      0.173     -2.310      0.021
   PREP1             -0.499      0.174     -2.868      0.004
   HELP3              0.214      0.124      1.726      0.084
   PREP4             -0.650      0.181     -3.589      0.000
   PREP3             -0.585      0.178     -3.293      0.001
   FPRB6              2.211      0.381      5.810      0.000
   HELP4              0.373      0.132      2.813      0.005

 PREP3    WITH
   PREP2              0.083      0.013      6.345      0.000

 PHPRB8   WITH
   PHPRB7             3.126      0.320      9.769      0.000
   PHPRB6             1.777      0.238      7.454      0.000
   FPRB6              1.205      0.277      4.348      0.000
   PHPRB5            -0.154      0.082     -1.877      0.060
   FPRB8              0.970      0.209      4.637      0.000
   FPRB10            -0.464      0.102     -4.567      0.000
   PREP4             -0.387      0.073     -5.267      0.000
   HELP2             -0.049      0.023     -2.117      0.034
```

PREP1	-0.275	0.070	-3.923	0.000
FPRB7	-0.265	0.128	-2.066	0.039
PREP2	-0.247	0.069	-3.564	0.000
FPRB5	0.724	0.250	2.894	0.004
**PHPRB9 WITH**				
FPRB8	1.088	0.208	5.217	0.000
PHPRB8	7.881	0.491	16.037	0.000
PREP3	0.319	0.065	4.887	0.000
PHPRB7	3.149	0.301	10.446	0.000
PHPRB6	2.016	0.225	8.976	0.000
FPRB6	1.477	0.257	5.750	0.000
FPRB9	0.342	0.099	3.464	0.001
FPRB5	0.791	0.236	3.359	0.001
**HELP1 WITH**				
PREP1	0.410	0.047	8.772	0.000
PREP2	0.319	0.044	7.303	0.000
**PHPRB3 WITH**				
PHPRB2	-0.014	0.048	-0.292	0.771
FPRB6	-0.330	0.080	-4.141	0.000
FPRB7	-0.245	0.104	-2.354	0.019
FPRB5	-0.107	0.054	-2.004	0.045
PREP2	-0.002	0.031	-0.063	0.950
PREP1	-0.021	0.031	-0.670	0.503
FPRB2	-0.184	0.059	-3.096	0.002
FPRB1	-0.164	0.059	-2.769	0.006
**PHPRB4 WITH**				
PREP3	-0.083	0.046	-1.790	0.073
PREP4	-0.033	0.049	-0.673	0.501
HELP3	0.011	0.054	0.196	0.845
FPRB8	0.063	0.100	0.624	0.532
FPRB10	0.458	0.110	4.173	0.000
FPRB9	0.201	0.102	1.976	0.048
PHPRB3	-1.968	0.414	-4.758	0.000
HELP2	0.020	0.018	1.064	0.288
PHPRB2	-1.453	0.318	-4.565	0.000
PHPRB1	-1.241	0.276	-4.490	0.000
FPRB7	0.105	0.174	0.605	0.545
FPRB6	-0.172	0.128	-1.341	0.180
HELP4	0.144	0.058	2.502	0.012
FPRB2	-0.204	0.100	-2.034	0.042
FPRB1	-0.162	0.100	-1.630	0.103
**FPRB8 WITH**				
FPRB5	2.034	0.228	8.933	0.000
FPRB4	-0.049	0.032	-1.561	0.119
PREP3	-0.280	0.130	-2.164	0.030
FPRB7	2.624	0.330	7.948	0.000
HELP4	0.644	0.118	5.469	0.000
HELP3	0.598	0.111	5.402	0.000
FPRB6	1.544	0.269	5.737	0.000
PREP1	-0.734	0.138	-5.334	0.000
PREP2	-0.652	0.136	-4.799	0.000
PREP4	-0.402	0.137	-2.940	0.003
**FPRB5 WITH**				
FPRB4	0.559	0.127	4.406	0.000
HELP3	0.065	0.095	0.684	0.494
FPRB3	0.476	0.123	3.876	0.000
PREP3	0.264	0.077	3.441	0.001
PREP4	0.161	0.086	1.875	0.061

HELP4	0.237	0.101	2.347	0.019
PREP1	-0.081	0.037	-2.165	0.030
PREP2 WITH				
PREP1	0.329	0.133	2.482	0.013
PHPRB1 WITH				
PREP1	-0.002	0.025	-0.082	0.934
FPRB7	-0.019	0.090	-0.216	0.829
PREP2	-0.004	0.027	-0.130	0.897
HELP3	-0.019	0.014	-1.406	0.160
FPRB5	0.060	0.033	1.816	0.069
HELP4 WITH				
HELP2	-0.157	0.061	-2.582	0.010
HELP3 WITH				
HELP1	0.123	0.060	2.051	0.040
FPRB6 WITH				
PREP2	0.283	0.089	3.165	0.002
PREP1	0.188	0.089	2.110	0.035
HELP3	0.032	0.111	0.286	0.775
HELP4	0.115	0.118	0.976	0.329
PREP3	0.129	0.059	2.205	0.027
FPRB5	1.318	0.246	5.347	0.000
Intercepts				
PREP1	2.721	0.091	29.771	0.000
PREP2	2.771	0.091	30.444	0.000
PREP3	3.064	0.099	30.940	0.000
PREP4	3.013	0.102	29.667	0.000
HELP1	3.727	0.110	34.013	0.000
HELP2	3.752	0.108	34.609	0.000
HELP3	4.043	0.110	36.727	0.000
HELP4	4.116	0.110	37.482	0.000
FPRB1	2.159	0.084	25.567	0.000
FPRB2	2.180	0.085	25.730	0.000
FPRB3	1.496	0.076	19.773	0.000
FPRB4	1.466	0.077	18.929	0.000
FPRB5	2.677	0.108	24.863	0.000
FPRB6	3.577	0.125	28.726	0.000
FPRB7	2.973	0.138	21.517	0.000
FPRB8	3.375	0.111	30.330	0.000
FPRB9	3.159	0.110	28.708	0.000
FPRB10	2.870	0.107	26.828	0.000
PHPRB1	0.446	0.048	9.310	0.000
PHPRB2	0.445	0.051	8.667	0.000
PHPRB3	0.561	0.051	11.028	0.000
PHPRB4	0.852	0.064	13.370	0.000
PHPRB5	1.436	0.080	17.858	0.000
PHPRB6	2.948	0.098	29.947	0.000
PHPRB7	3.239	0.108	29.890	0.000
PHPRB8	5.775	0.129	44.698	0.000
PHPRB9	5.714	0.122	46.653	0.000
PHPRB10	5.911	0.124	47.594	0.000
Variances				
PREP	4.234	0.300	14.133	0.000
FPRB	2.807	0.276	10.185	0.000
Residual Variances				
PREP1	0.446	0.135	3.311	0.001
PREP2	0.429	0.133	3.222	0.001

PREP3	0.628	0.155	4.044	0.000
PREP4	0.650	0.165	3.943	0.000
HELP1	1.293	0.098	13.172	0.000
HELP2	0.937	0.091	10.264	0.000
HELP3	0.410	0.074	5.512	0.000
HELP4	0.299	0.079	3.799	0.000
FPRB1	1.186	0.180	6.592	0.000
FPRB2	1.102	0.182	6.070	0.000
FPRB3	1.927	0.133	14.498	0.000
FPRB4	2.045	0.140	14.630	0.000
FPRB5	3.789	0.275	13.754	0.000
FPRB6	4.526	0.371	12.189	0.000
FPRB7	5.394	0.598	9.026	0.000
FPRB8	5.317	0.347	15.323	0.000
FPRB9	4.863	0.324	15.019	0.000
FPRB10	4.955	0.394	12.585	0.000
PHPRB1	0.570	0.061	9.337	0.000
PHPRB2	0.592	0.085	6.995	0.000
PHPRB3	-0.070	0.106	-0.659	0.510
PHPRB4	-4.476	1.585	-2.824	0.005
PHPRB5	2.990	0.176	16.957	0.000
PHPRB6	4.915	0.258	19.084	0.000
PHPRB7	6.385	0.327	19.508	0.000
PHPRB8	9.239	0.559	16.526	0.000
PHPRB9	8.299	0.484	17.147	0.000
PHPRB10	8.548	0.499	17.116	0.000
HELP	4.320	0.307	14.089	0.000
PHPRB	0.662	0.079	8.391	0.000

STANDARDIZED MODEL RESULTS

STDYX Standardization

	Estimate	S.E.	Est./S.E.	Two-Tailed P-Value
PREP BY				
PREP1	0.951	0.015	62.196	0.000
PREP2	0.953	0.015	62.548	0.000
PREP3	0.941	0.015	61.297	0.000
PREP4	0.942	0.015	60.939	0.000
HELP BY				
HELP1	0.899	0.010	93.792	0.000
HELP2	0.926	0.008	110.755	0.000
HELP3	0.969	0.006	163.350	0.000
HELP4	0.978	0.006	159.332	0.000
FPRB BY				
FPRB1	0.838	0.028	30.338	0.000
FPRB2	0.852	0.027	31.334	0.000
FPRB3	0.632	0.030	21.065	0.000
FPRB4	0.626	0.030	20.695	0.000
FPRB5	0.645	0.031	20.577	0.000
FPRB6	0.692	0.030	22.954	0.000
FPRB7	0.704	0.038	18.324	0.000
FPRB8	0.483	0.036	13.593	0.000
FPRB9	0.532	0.033	16.316	0.000
FPRB10	0.476	0.053	8.905	0.000
PHPRB BY				
PHPRB1	0.747	0.032	23.174	0.000

```
    PHPRB2             0.774      0.038     20.583      0.000
    PHPRB3             1.024      0.036     28.635      0.000
    PHPRB4             1.723      0.208      8.291      0.000
    PHPRB5             0.417      0.035     11.940      0.000
    PHPRB6             0.307      0.035      8.831      0.000
    PHPRB7             0.171      0.035      4.943      0.000
    PHPRB8            -0.108      0.028     -3.895      0.000
    PHPRB9            -0.110      0.028     -4.004      0.000
    PHPRB10           -0.101      0.027     -3.813      0.000

 HELP     ON
    PREP               0.452      0.035     13.011      0.000

 PHPRB    ON
    PREP              -0.008      0.028     -0.303      0.762
    FPRB               0.287      0.048      5.987      0.000
    HELP              -0.029      0.032     -0.886      0.376

 FPRB     WITH
    HELP               0.444      0.037     12.140      0.000
    PREP              -0.016      0.045     -0.351      0.726

 FPRB2    WITH
    FPRB1              0.908      0.015     59.870      0.000
    HELP1             -0.019      0.008     -2.509      0.012

 FPRB4    WITH
    FPRB3              0.921      0.007    133.199      0.000
    PREP3              0.019      0.012      1.531      0.126

 PHPRB10  WITH
    PHPRB9             0.903      0.008    116.597      0.000
    PHPRB8             0.873      0.010     90.250      0.000
    FPRB8              0.101      0.031      3.278      0.001
    PHPRB7             0.418      0.033     12.608      0.000
    PHPRB6             0.317      0.031     10.109      0.000
    FPRB5              0.118      0.042      2.819      0.005
    PHPRB3           999.000    999.000    999.000    999.000
    HELP4             -0.038      0.025     -1.518      0.129
    FPRB6              0.255      0.041      6.216      0.000
    FPRB9              0.045      0.016      2.754      0.006
    PHPRB2            -0.015      0.010     -1.491      0.136
    PREP3              0.108      0.030      3.567      0.000
    FPRB2              0.078      0.025      3.110      0.002
    FPRB1              0.068      0.024      2.801      0.005

 PHPRB7   WITH
    PHPRB6             0.823      0.013     61.363      0.000
    PHPRB5             0.435      0.031     13.905      0.000
    FPRB9              0.451      0.033     13.547      0.000
    PHPRB4           999.000    999.000    999.000    999.000
    FPRB10             0.303      0.036      8.410      0.000
    FPRB5              0.232      0.039      5.945      0.000
    FPRB8              0.372      0.035     10.563      0.000
    PHPRB1            -0.041      0.014     -2.986      0.003
    PREP4              0.332      0.062      5.371      0.000
    PREP3              0.338      0.060      5.610      0.000
    FPRB7              0.199      0.043      4.656      0.000
    HELP4              0.462      0.098      4.696      0.000
    HELP3              0.310      0.073      4.271      0.000
    FPRB6              0.115      0.045      2.571      0.010

 PREP4    WITH
    PREP3              0.644      0.089      7.219      0.000
```

```
 HELP2    WITH
    HELP1              0.910      0.011     79.273      0.000
    PREP1              0.601      0.098      6.166      0.000
    PREP2              0.518      0.090      5.790      0.000

 PHPRB2   WITH
    PHPRB1             0.809      0.023     35.718      0.000
    FPRB2             -0.130      0.047     -2.778      0.005
    FPRB9             -0.043      0.011     -3.727      0.000
    FPRB7             -0.125      0.058     -2.158      0.031
    PREP4              0.110      0.054      2.044      0.041
    HELP2             -0.018      0.012     -1.550      0.121
    PREP2              0.032      0.037      0.852      0.394
    FPRB6             -0.076      0.030     -2.505      0.012
    PREP3              0.081      0.052      1.559      0.119
    FPRB1             -0.084      0.043     -1.938      0.053

 FPRB9    WITH
    FPRB8              0.778      0.018     43.837      0.000
    FPRB5              0.399      0.038     10.539      0.000
    PREP2             -0.304      0.063     -4.795      0.000
    PREP1             -0.346      0.067     -5.183      0.000
    FPRB7              0.536      0.035     15.275      0.000
    HELP4              0.621      0.113      5.480      0.000
    HELP3              0.456      0.080      5.701      0.000
    FPRB6              0.312      0.047      6.622      0.000

 FPRB10   WITH
    FPRB9              0.810      0.017     48.794      0.000
    FPRB8              0.638      0.026     24.713      0.000
    HELP3              0.302      0.074      4.074      0.000
    FPRB3              0.167      0.040      4.214      0.000
    FPRB5              0.423      0.047      9.040      0.000
    FPRB4              0.147      0.040      3.719      0.000
    FPRB2              0.170      0.081      2.090      0.037
    FPRB7              0.593      0.044     13.349      0.000
    FPRB6              0.393      0.056      7.058      0.000
    FPRB1              0.183      0.077      2.374      0.018
    HELP4              0.495      0.101      4.905      0.000
    PREP1             -0.252      0.060     -4.179      0.000
    PREP2             -0.198      0.057     -3.500      0.000

 PHPRB5   WITH
    PHPRB4           999.000    999.000    999.000    999.000
    FPRB3              0.320      0.039      8.245      0.000
    FPRB9              0.524      0.037     14.057      0.000
    FPRB4              0.286      0.039      7.366      0.000
    FPRB10             0.546      0.036     15.221      0.000
    FPRB8              0.415      0.038     10.835      0.000
    FPRB5              0.396      0.045      8.729      0.000
    FPRB6              0.326      0.053      6.162      0.000
    FPRB7              0.445      0.052      8.591      0.000
    PREP2             -0.340      0.071     -4.820      0.000
    PREP1             -0.331      0.069     -4.794      0.000
    FPRB1              0.389      0.071      5.476      0.000
    FPRB2              0.381      0.074      5.147      0.000
    HELP4              0.518      0.106      4.875      0.000
    HELP3              0.322      0.078      4.126      0.000

 PHPRB6   WITH
    PHPRB5             0.579      0.026     22.557      0.000
    FPRB9              0.580      0.031     18.883      0.000
    PHPRB4           999.000    999.000    999.000    999.000
```

FPRB2	0.199	0.041	4.892	0.000
FPRB10	0.437	0.034	12.774	0.000
FPRB8	0.403	0.035	11.409	0.000
PREP4	0.417	0.068	6.163	0.000
PREP3	0.399	0.064	6.238	0.000
FPRB5	0.258	0.039	6.563	0.000
FPRB1	0.169	0.039	4.367	0.000
FPRB6	0.215	0.046	4.692	0.000
FPRB7	0.315	0.044	7.104	0.000
HELP4	0.588	0.110	5.349	0.000
HELP3	0.371	0.079	4.696	0.000
PHPRB3	999.000	999.000	999.000	999.000
FPRB7 WITH				
FPRB5	0.206	0.052	3.952	0.000
FPRB2	-0.005	0.084	-0.059	0.953
FPRB1	0.015	0.078	0.197	0.844
PREP2	-0.263	0.119	-2.219	0.026
PREP1	-0.322	0.119	-2.707	0.007
HELP3	0.144	0.082	1.764	0.078
PREP4	-0.347	0.100	-3.467	0.001
PREP3	-0.318	0.098	-3.230	0.001
FPRB6	0.448	0.048	9.285	0.000
HELP4	0.293	0.103	2.857	0.004
PREP3 WITH				
PREP2	0.159	0.023	6.833	0.000
PHPRB8 WITH				
PHPRB7	0.407	0.033	12.222	0.000
PHPRB6	0.264	0.033	8.061	0.000
FPRB6	0.186	0.042	4.400	0.000
PHPRB5	-0.029	0.016	-1.890	0.059
FPRB8	0.138	0.029	4.760	0.000
FPRB10	-0.069	0.014	-4.751	0.000
PREP4	-0.158	0.034	-4.597	0.000
HELP2	-0.017	0.008	-2.093	0.036
PREP1	-0.136	0.039	-3.496	0.000
FPRB7	-0.038	0.018	-2.093	0.036
PREP2	-0.124	0.038	-3.225	0.001
FPRB5	0.122	0.042	2.936	0.003
PHPRB9 WITH				
FPRB8	0.164	0.031	5.326	0.000
PHPRB8	0.900	0.008	116.384	0.000
PREP3	0.140	0.032	4.303	0.000
PHPRB7	0.433	0.033	13.243	0.000
PHPRB6	0.316	0.031	10.026	0.000
FPRB6	0.241	0.041	5.943	0.000
FPRB9	0.054	0.016	3.451	0.001
FPRB5	0.141	0.041	3.413	0.001
HELP1 WITH				
PREP1	0.541	0.089	6.074	0.000
PREP2	0.428	0.078	5.471	0.000
PHPRB3 WITH				
PHPRB2	999.000	999.000	999.000	999.000
FPRB6	999.000	999.000	999.000	999.000
FPRB7	999.000	999.000	999.000	999.000
FPRB5	999.000	999.000	999.000	999.000
PREP2	999.000	999.000	999.000	999.000
PREP1	999.000	999.000	999.000	999.000
FPRB2	999.000	999.000	999.000	999.000

FPRB1	999.000	999.000	999.000	999.000
**PHPRB4 WITH**				
PREP3	999.000	999.000	999.000	999.000
PREP4	999.000	999.000	999.000	999.000
HELP3	999.000	999.000	999.000	999.000
FPRB8	999.000	999.000	999.000	999.000
FPRB10	999.000	999.000	999.000	999.000
FPRB9	999.000	999.000	999.000	999.000
PHPRB3	999.000	999.000	999.000	999.000
HELP2	999.000	999.000	999.000	999.000
PHPRB2	999.000	999.000	999.000	999.000
PHPRB1	999.000	999.000	999.000	999.000
FPRB7	999.000	999.000	999.000	999.000
FPRB6	999.000	999.000	999.000	999.000
HELP4	999.000	999.000	999.000	999.000
FPRB2	999.000	999.000	999.000	999.000
FPRB1	999.000	999.000	999.000	999.000
**FPRB8 WITH**				
FPRB5	0.453	0.035	13.118	0.000
FPRB4	-0.015	0.010	-1.559	0.119
PREP3	-0.153	0.071	-2.150	0.032
FPRB7	0.490	0.037	13.368	0.000
HELP4	0.511	0.106	4.818	0.000
HELP3	0.405	0.076	5.325	0.000
FPRB6	0.315	0.044	7.125	0.000
PREP1	-0.477	0.104	-4.575	0.000
PREP2	-0.431	0.102	-4.211	0.000
PREP4	-0.216	0.075	-2.868	0.004
**FPRB5 WITH**				
FPRB4	0.201	0.041	4.948	0.000
HELP3	0.052	0.076	0.683	0.494
FPRB3	0.176	0.041	4.272	0.000
PREP3	0.171	0.054	3.147	0.002
PREP4	0.103	0.057	1.805	0.071
HELP4	0.223	0.096	2.322	0.020
PREP1	-0.062	0.030	-2.089	0.037
**PREP2 WITH**				
PREP1	0.753	0.076	9.932	0.000
**PHPRB1 WITH**				
PREP1	-0.004	0.050	-0.082	0.935
FPRB7	-0.011	0.051	-0.215	0.830
PREP2	-0.007	0.055	-0.130	0.897
HELP3	-0.040	0.029	-1.400	0.161
FPRB5	0.041	0.023	1.809	0.070
**HELP4 WITH**				
HELP2	-0.296	0.156	-1.897	0.058
**HELP3 WITH**				
HELP1	0.169	0.067	2.524	0.012
**FPRB6 WITH**				
PREP2	0.203	0.070	2.914	0.004
PREP1	0.132	0.065	2.030	0.042
HELP3	0.023	0.081	0.287	0.774
HELP4	0.099	0.100	0.984	0.325
PREP3	0.077	0.036	2.118	0.034
FPRB5	0.318	0.046	6.914	0.000

Intercepts				
PREP1	1.258	0.056	22.495	0.000
PREP2	1.286	0.057	22.762	0.000
PREP3	1.307	0.057	23.033	0.000
PREP4	1.254	0.055	22.600	0.000
HELP1	1.437	0.060	23.939	0.000
HELP2	1.462	0.061	24.118	0.000
HELP3	1.552	0.063	24.714	0.000
HELP4	1.584	0.063	25.054	0.000
FPRB1	1.080	0.052	20.581	0.000
FPRB2	1.087	0.052	20.723	0.000
FPRB3	0.836	0.049	17.056	0.000
FPRB4	0.800	0.048	16.501	0.000
FPRB5	1.051	0.052	20.303	0.000
FPRB6	1.214	0.054	22.414	0.000
FPRB7	0.909	0.049	18.414	0.000
FPRB8	1.282	0.056	22.960	0.000
FPRB9	1.213	0.053	22.685	0.000
FPRB10	1.134	0.052	21.626	0.000
PHPRB1	0.393	0.044	8.970	0.000
PHPRB2	0.366	0.044	8.396	0.000
PHPRB3	0.466	0.044	10.485	0.000
PHPRB4	0.565	0.045	12.460	0.000
PHPRB5	0.755	0.047	15.920	0.000
PHPRB6	1.266	0.053	23.819	0.000
PHPRB7	1.263	0.053	23.707	0.000
PHPRB8	1.889	0.071	26.496	0.000
PHPRB9	1.971	0.072	27.528	0.000
PHPRB10	2.011	0.073	27.681	0.000
Variances				
PREP	1.000	0.000	999.000	999.000
FPRB	1.000	0.000	999.000	999.000
Residual Variances				
PREP1	0.095	0.029	3.273	0.001
PREP2	0.092	0.029	3.186	0.001
PREP3	0.114	0.029	3.959	0.000
PREP4	0.113	0.029	3.867	0.000
HELP1	0.192	0.017	11.163	0.000
HELP2	0.142	0.015	9.189	0.000
HELP3	0.060	0.012	5.257	0.000
HELP4	0.044	0.012	3.696	0.000
FPRB1	0.297	0.046	6.409	0.000
FPRB2	0.274	0.046	5.913	0.000
FPRB3	0.601	0.038	15.847	0.000
FPRB4	0.609	0.038	16.093	0.000
FPRB5	0.584	0.040	14.424	0.000
FPRB6	0.521	0.042	12.497	0.000
FPRB7	0.505	0.054	9.329	0.000
FPRB8	0.767	0.034	22.348	0.000
FPRB9	0.717	0.035	20.691	0.000
FPRB10	0.773	0.051	15.188	0.000
PHPRB1	0.442	0.048	9.195	0.000
PHPRB2	0.401	0.058	6.902	0.000
PHPRB3	-0.048	999.000	999.000	999.000
PHPRB4	-1.969	999.000	999.000	999.000
PHPRB5	0.826	0.029	28.340	0.000
PHPRB6	0.906	0.021	42.398	0.000
PHPRB7	0.971	0.012	82.171	0.000
PHPRB8	0.988	0.006	164.732	0.000
PHPRB9	0.988	0.006	162.164	0.000
PHPRB10	0.990	0.005	183.754	0.000
HELP	0.795	0.031	25.299	0.000

	Estimate	S.E.	Est./S.E.	Two-Tailed P-Value
PHPRB	0.923	0.024	38.123	0.000

STDY Standardization

	Estimate	S.E.	Est./S.E.	Two-Tailed P-Value
**PREP BY**				
PREP1	0.951	0.015	62.196	0.000
PREP2	0.953	0.015	62.548	0.000
PREP3	0.941	0.015	61.297	0.000
PREP4	0.942	0.015	60.939	0.000
**HELP BY**				
HELP1	0.899	0.010	93.792	0.000
HELP2	0.926	0.008	110.755	0.000
HELP3	0.969	0.006	163.350	0.000
HELP4	0.978	0.006	159.332	0.000
**FPRB BY**				
FPRB1	0.838	0.028	30.338	0.000
FPRB2	0.852	0.027	31.334	0.000
FPRB3	0.632	0.030	21.065	0.000
FPRB4	0.626	0.030	20.695	0.000
FPRB5	0.645	0.031	20.577	0.000
FPRB6	0.692	0.030	22.954	0.000
FPRB7	0.704	0.038	18.324	0.000
FPRB8	0.483	0.036	13.593	0.000
FPRB9	0.532	0.033	16.316	0.000
FPRB10	0.476	0.053	8.905	0.000
**PHPRB BY**				
PHPRB1	0.747	0.032	23.174	0.000
PHPRB2	0.774	0.038	20.583	0.000
PHPRB3	1.024	0.036	28.635	0.000
PHPRB4	1.723	0.208	8.291	0.000
PHPRB5	0.417	0.035	11.940	0.000
PHPRB6	0.307	0.035	8.831	0.000
PHPRB7	0.171	0.035	4.943	0.000
PHPRB8	-0.108	0.028	-3.895	0.000
PHPRB9	-0.110	0.028	-4.004	0.000
PHPRB10	-0.101	0.027	-3.813	0.000
**HELP ON**				
PREP	0.452	0.035	13.011	0.000
**PHPRB ON**				
PREP	-0.008	0.028	-0.303	0.762
FPRB	0.287	0.048	5.987	0.000
HELP	-0.029	0.032	-0.886	0.376
**FPRB WITH**				
HELP	0.444	0.037	12.140	0.000
PREP	-0.016	0.045	-0.351	0.726
**FPRB2 WITH**				
FPRB1	0.908	0.015	59.870	0.000
HELP1	-0.019	0.008	-2.509	0.012
**FPRB4 WITH**				
FPRB3	0.921	0.007	133.199	0.000
PREP3	0.019	0.012	1.531	0.126

PHPRB10 WITH				
PHPRB9	0.903	0.008	116.597	0.000
PHPRB8	0.873	0.010	90.250	0.000
FPRB8	0.101	0.031	3.278	0.001
PHPRB7	0.418	0.033	12.608	0.000
PHPRB6	0.317	0.031	10.109	0.000
FPRB5	0.118	0.042	2.819	0.005
PHPRB3	999.000	999.000	999.000	999.000
HELP4	-0.038	0.025	-1.518	0.129
FPRB6	0.255	0.041	6.216	0.000
FPRB9	0.045	0.016	2.754	0.006
PHPRB2	-0.015	0.010	-1.491	0.136
PREP3	0.108	0.030	3.567	0.000
FPRB2	0.078	0.025	3.110	0.002
FPRB1	0.068	0.024	2.801	0.005
PHPRB7 WITH				
PHPRB6	0.823	0.013	61.363	0.000
PHPRB5	0.435	0.031	13.905	0.000
FPRB9	0.451	0.033	13.547	0.000
PHPRB4	999.000	999.000	999.000	999.000
FPRB10	0.303	0.036	8.410	0.000
FPRB5	0.232	0.039	5.945	0.000
FPRB8	0.372	0.035	10.563	0.000
PHPRB1	-0.041	0.014	-2.986	0.003
PREP4	0.332	0.062	5.371	0.000
PREP3	0.338	0.060	5.610	0.000
FPRB7	0.199	0.043	4.656	0.000
HELP4	0.462	0.098	4.696	0.000
HELP3	0.310	0.073	4.271	0.000
FPRB6	0.115	0.045	2.571	0.010
PREP4 WITH				
PREP3	0.644	0.089	7.219	0.000
HELP2 WITH				
HELP1	0.910	0.011	79.273	0.000
PREP1	0.601	0.098	6.166	0.000
PREP2	0.518	0.090	5.790	0.000
PHPRB2 WITH				
PHPRB1	0.809	0.023	35.718	0.000
FPRB2	-0.130	0.047	-2.778	0.005
FPRB9	-0.043	0.011	-3.727	0.000
FPRB7	-0.125	0.058	-2.158	0.031
PREP4	0.110	0.054	2.044	0.041
HELP2	-0.018	0.012	-1.550	0.121
PREP2	0.032	0.037	0.852	0.394
FPRB6	-0.076	0.030	-2.505	0.012
PREP3	0.081	0.052	1.559	0.119
FPRB1	-0.084	0.043	-1.938	0.053
FPRB9 WITH				
FPRB8	0.778	0.018	43.837	0.000
FPRB5	0.399	0.038	10.539	0.000
PREP2	-0.304	0.063	-4.795	0.000
PREP1	-0.346	0.067	-5.183	0.000
FPRB7	0.536	0.035	15.275	0.000
HELP4	0.621	0.113	5.480	0.000
HELP3	0.456	0.080	5.701	0.000
FPRB6	0.312	0.047	6.622	0.000
FPRB10 WITH				
FPRB9	0.810	0.017	48.794	0.000

```
   FPRB8              0.638      0.026     24.713      0.000
   HELP3              0.302      0.074      4.074      0.000
   FPRB3              0.167      0.040      4.214      0.000
   FPRB5              0.423      0.047      9.040      0.000
   FPRB4              0.147      0.040      3.719      0.000
   FPRB2              0.170      0.081      2.090      0.037
   FPRB7              0.593      0.044     13.349      0.000
   FPRB6              0.393      0.056      7.058      0.000
   FPRB1              0.183      0.077      2.374      0.018
   HELP4              0.495      0.101      4.905      0.000
   PREP1             -0.252      0.060     -4.179      0.000
   PREP2             -0.198      0.057     -3.500      0.000

 PHPRB5   WITH
   PHPRB4           999.000    999.000    999.000    999.000
   FPRB3              0.320      0.039      8.245      0.000
   FPRB9              0.524      0.037     14.057      0.000
   FPRB4              0.286      0.039      7.366      0.000
   FPRB10             0.546      0.036     15.221      0.000
   FPRB8              0.415      0.038     10.835      0.000
   FPRB5              0.396      0.045      8.729      0.000
   FPRB6              0.326      0.053      6.162      0.000
   FPRB7              0.445      0.052      8.591      0.000
   PREP2             -0.340      0.071     -4.820      0.000
   PREP1             -0.331      0.069     -4.794      0.000
   FPRB1              0.389      0.071      5.476      0.000
   FPRB2              0.381      0.074      5.147      0.000
   HELP4              0.518      0.106      4.875      0.000
   HELP3              0.322      0.078      4.126      0.000

 PHPRB6   WITH
   PHPRB5             0.579      0.026     22.557      0.000
   FPRB9              0.580      0.031     18.883      0.000
   PHPRB4           999.000    999.000    999.000    999.000
   FPRB2              0.199      0.041      4.892      0.000
   FPRB10             0.437      0.034     12.774      0.000
   FPRB8              0.403      0.035     11.409      0.000
   PREP4              0.417      0.068      6.163      0.000
   PREP3              0.399      0.064      6.238      0.000
   FPRB5              0.258      0.039      6.563      0.000
   FPRB1              0.169      0.039      4.367      0.000
   FPRB6              0.215      0.046      4.692      0.000
   FPRB7              0.315      0.044      7.104      0.000
   HELP4              0.588      0.110      5.349      0.000
   HELP3              0.371      0.079      4.696      0.000
   PHPRB3           999.000    999.000    999.000    999.000

 FPRB7    WITH
   FPRB5              0.206      0.052      3.952      0.000
   FPRB2             -0.005      0.084     -0.059      0.953
   FPRB1              0.015      0.078      0.197      0.844
   PREP2             -0.263      0.119     -2.219      0.026
   PREP1             -0.322      0.119     -2.707      0.007
   HELP3              0.144      0.082      1.764      0.078
   PREP4             -0.347      0.100     -3.467      0.001
   PREP3             -0.318      0.098     -3.230      0.001
   FPRB6              0.448      0.048      9.285      0.000
   HELP4              0.293      0.103      2.857      0.004

 PREP3    WITH
   PREP2              0.159      0.023      6.833      0.000

 PHPRB8   WITH
   PHPRB7             0.407      0.033     12.222      0.000
```

PHPRB6	0.264	0.033	8.061	0.000
FPRB6	0.186	0.042	4.400	0.000
PHPRB5	-0.029	0.016	-1.890	0.059
FPRB8	0.138	0.029	4.760	0.000
FPRB10	-0.069	0.014	-4.751	0.000
PREP4	-0.158	0.034	-4.597	0.000
HELP2	-0.017	0.008	-2.093	0.036
PREP1	-0.136	0.039	-3.496	0.000
FPRB7	-0.038	0.018	-2.093	0.036
PREP2	-0.124	0.038	-3.225	0.001
FPRB5	0.122	0.042	2.936	0.003
**PHPRB9 WITH**				
FPRB8	0.164	0.031	5.326	0.000
PHPRB8	0.900	0.008	116.384	0.000
PREP3	0.140	0.032	4.303	0.000
PHPRB7	0.433	0.033	13.243	0.000
PHPRB6	0.316	0.031	10.026	0.000
FPRB6	0.241	0.041	5.943	0.000
FPRB9	0.054	0.016	3.451	0.001
FPRB5	0.141	0.041	3.413	0.001
**HELP1 WITH**				
PREP1	0.541	0.089	6.074	0.000
PREP2	0.428	0.078	5.471	0.000
**PHPRB3 WITH**				
PHPRB2	999.000	999.000	999.000	999.000
FPRB6	999.000	999.000	999.000	999.000
FPRB7	999.000	999.000	999.000	999.000
FPRB5	999.000	999.000	999.000	999.000
PREP2	999.000	999.000	999.000	999.000
PREP1	999.000	999.000	999.000	999.000
FPRB2	999.000	999.000	999.000	999.000
FPRB1	999.000	999.000	999.000	999.000
**PHPRB4 WITH**				
PREP3	999.000	999.000	999.000	999.000
PREP4	999.000	999.000	999.000	999.000
HELP3	999.000	999.000	999.000	999.000
FPRB8	999.000	999.000	999.000	999.000
FPRB10	999.000	999.000	999.000	999.000
FPRB9	999.000	999.000	999.000	999.000
PHPRB3	999.000	999.000	999.000	999.000
HELP2	999.000	999.000	999.000	999.000
PHPRB2	999.000	999.000	999.000	999.000
PHPRB1	999.000	999.000	999.000	999.000
FPRB7	999.000	999.000	999.000	999.000
FPRB6	999.000	999.000	999.000	999.000
HELP4	999.000	999.000	999.000	999.000
FPRB2	999.000	999.000	999.000	999.000
FPRB1	999.000	999.000	999.000	999.000
**FPRB8 WITH**				
FPRB5	0.453	0.035	13.118	0.000
FPRB4	-0.015	0.010	-1.559	0.119
PREP3	-0.153	0.071	-2.150	0.032
FPRB7	0.490	0.037	13.368	0.000
HELP4	0.511	0.106	4.818	0.000
HELP3	0.405	0.076	5.325	0.000
FPRB6	0.315	0.044	7.125	0.000
PREP1	-0.477	0.104	-4.575	0.000
PREP2	-0.431	0.102	-4.211	0.000
PREP4	-0.216	0.075	-2.868	0.004

```
 FPRB5    WITH
    FPRB4              0.201      0.041      4.948      0.000
    HELP3              0.052      0.076      0.683      0.494
    FPRB3              0.176      0.041      4.272      0.000
    PREP3              0.171      0.054      3.147      0.002
    PREP4              0.103      0.057      1.805      0.071
    HELP4              0.223      0.096      2.322      0.020
    PREP1             -0.062      0.030     -2.089      0.037

 PREP2    WITH
    PREP1              0.753      0.076      9.932      0.000

 PHPRB1   WITH
    PREP1             -0.004      0.050     -0.082      0.935
    FPRB7             -0.011      0.051     -0.215      0.830
    PREP2             -0.007      0.055     -0.130      0.897
    HELP3             -0.040      0.029     -1.400      0.161
    FPRB5              0.041      0.023      1.809      0.070

 HELP4    WITH
    HELP2             -0.296      0.156     -1.897      0.058

 HELP3    WITH
    HELP1              0.169      0.067      2.524      0.012

 FPRB6    WITH
    PREP2              0.203      0.070      2.914      0.004
    PREP1              0.132      0.065      2.030      0.042
    HELP3              0.023      0.081      0.287      0.774
    HELP4              0.099      0.100      0.984      0.325
    PREP3              0.077      0.036      2.118      0.034
    FPRB5              0.318      0.046      6.914      0.000

 Intercepts
    PREP1              1.258      0.056     22.495      0.000
    PREP2              1.286      0.057     22.762      0.000
    PREP3              1.307      0.057     23.033      0.000
    PREP4              1.254      0.055     22.600      0.000
    HELP1              1.437      0.060     23.939      0.000
    HELP2              1.462      0.061     24.118      0.000
    HELP3              1.552      0.063     24.714      0.000
    HELP4              1.584      0.063     25.054      0.000
    FPRB1              1.080      0.052     20.581      0.000
    FPRB2              1.087      0.052     20.723      0.000
    FPRB3              0.836      0.049     17.056      0.000
    FPRB4              0.800      0.048     16.501      0.000
    FPRB5              1.051      0.052     20.303      0.000
    FPRB6              1.214      0.054     22.414      0.000
    FPRB7              0.909      0.049     18.414      0.000
    FPRB8              1.282      0.056     22.960      0.000
    FPRB9              1.213      0.053     22.685      0.000
    FPRB10             1.134      0.052     21.626      0.000
    PHPRB1             0.393      0.044      8.970      0.000
    PHPRB2             0.366      0.044      8.396      0.000
    PHPRB3             0.466      0.044     10.485      0.000
    PHPRB4             0.565      0.045     12.460      0.000
    PHPRB5             0.755      0.047     15.920      0.000
    PHPRB6             1.266      0.053     23.819      0.000
    PHPRB7             1.263      0.053     23.707      0.000
    PHPRB8             1.889      0.071     26.496      0.000
    PHPRB9             1.971      0.072     27.528      0.000
    PHPRB10            2.011      0.073     27.681      0.000
```

	Estimate	S.E.	Est./S.E.	Two-Tailed P-Value
**Variances**				
PREP	1.000	0.000	999.000	999.000
FPRB	1.000	0.000	999.000	999.000
**Residual Variances**				
PREP1	0.095	0.029	3.273	0.001
PREP2	0.092	0.029	3.186	0.001
PREP3	0.114	0.029	3.959	0.000
PREP4	0.113	0.029	3.867	0.000
HELP1	0.192	0.017	11.163	0.000
HELP2	0.142	0.015	9.189	0.000
HELP3	0.060	0.012	5.257	0.000
HELP4	0.044	0.012	3.696	0.000
FPRB1	0.297	0.046	6.409	0.000
FPRB2	0.274	0.046	5.913	0.000
FPRB3	0.601	0.038	15.847	0.000
FPRB4	0.609	0.038	16.093	0.000
FPRB5	0.584	0.040	14.424	0.000
FPRB6	0.521	0.042	12.497	0.000
FPRB7	0.505	0.054	9.329	0.000
FPRB8	0.767	0.034	22.348	0.000
FPRB9	0.717	0.035	20.691	0.000
FPRB10	0.773	0.051	15.188	0.000
PHPRB1	0.442	0.048	9.195	0.000
PHPRB2	0.401	0.058	6.902	0.000
PHPRB3	999.000	999.000	999.000	999.000
PHPRB4	999.000	999.000	999.000	999.000
PHPRB5	0.826	0.029	28.340	0.000
PHPRB6	0.906	0.021	42.398	0.000
PHPRB7	0.971	0.012	82.171	0.000
PHPRB8	0.988	0.006	164.732	0.000
PHPRB9	0.988	0.006	162.164	0.000
PHPRB10	0.990	0.005	183.754	0.000
HELP	0.795	0.031	25.299	0.000
PHPRB	0.923	0.024	38.123	0.000

STD Standardization

	Estimate	S.E.	Est./S.E.	Two-Tailed P-Value
**PREP BY**				
PREP1	2.058	0.073	28.266	0.000
PREP2	2.052	0.073	28.301	0.000
PREP3	2.206	0.080	27.726	0.000
PREP4	2.264	0.081	28.001	0.000
**HELP BY**				
HELP1	2.330	0.085	27.333	0.000
HELP2	2.376	0.083	28.748	0.000
HELP3	2.525	0.082	30.952	0.000
HELP4	2.540	0.081	31.516	0.000
**FPRB BY**				
FPRB1	1.675	0.082	20.370	0.000
FPRB2	1.709	0.082	20.927	0.000
FPRB3	1.132	0.073	15.494	0.000
FPRB4	1.147	0.075	15.300	0.000
FPRB5	1.644	0.106	15.551	0.000
FPRB6	2.039	0.119	17.062	0.000
FPRB7	2.302	0.152	15.124	0.000
FPRB8	1.272	0.107	11.927	0.000
FPRB9	1.385	0.098	14.132	0.000

FPRB10	1.205	0.144	8.384	0.000
**PHPRB BY**				
PHPRB1	0.847	0.050	16.831	0.000
PHPRB2	0.939	0.059	15.980	0.000
PHPRB3	1.232	0.056	22.194	0.000
PHPRB4	2.598	0.304	8.547	0.000
PHPRB5	0.794	0.075	10.529	0.000
PHPRB6	0.715	0.086	8.324	0.000
PHPRB7	0.438	0.091	4.827	0.000
PHPRB8	-0.330	0.086	-3.825	0.000
PHPRB9	-0.320	0.081	-3.928	0.000
PHPRB10	-0.298	0.080	-3.744	0.000
**HELP ON**				
PREP	0.452	0.035	13.011	0.000
**PHPRB ON**				
PREP	-0.008	0.028	-0.303	0.762
FPRB	0.287	0.048	5.987	0.000
HELP	-0.029	0.032	-0.886	0.376
**FPRB WITH**				
HELP	0.444	0.037	12.140	0.000
PREP	-0.016	0.045	-0.351	0.726
**FPRB2 WITH**				
FPRB1	1.038	0.178	5.828	0.000
HELP1	-0.023	0.009	-2.565	0.010
**FPRB4 WITH**				
FPRB3	1.828	0.132	13.849	0.000
PREP3	0.021	0.013	1.564	0.118
**PHPRB10 WITH**				
PHPRB9	7.603	0.468	16.231	0.000
PHPRB8	7.760	0.491	15.789	0.000
FPRB8	0.681	0.208	3.284	0.001
PHPRB7	3.085	0.307	10.054	0.000
PHPRB6	2.054	0.228	8.999	0.000
FPRB5	0.670	0.239	2.800	0.005
PHPRB3	999.000	999.000	999.000	999.000
HELP4	-0.060	0.040	-1.517	0.129
FPRB6	1.585	0.262	6.052	0.000
FPRB9	0.289	0.104	2.772	0.006
PHPRB2	-0.034	0.023	-1.498	0.134
PREP3	0.251	0.066	3.824	0.000
FPRB2	0.241	0.077	3.140	0.002
FPRB1	0.216	0.077	2.814	0.005
**PHPRB7 WITH**				
PHPRB6	4.611	0.260	17.703	0.000
PHPRB5	1.899	0.182	10.419	0.000
FPRB9	2.515	0.240	10.468	0.000
PHPRB4	999.000	999.000	999.000	999.000
FPRB10	1.707	0.224	7.632	0.000
FPRB5	1.139	0.201	5.661	0.000
FPRB8	2.166	0.240	9.015	0.000
PHPRB1	-0.079	0.026	-2.987	0.003
PREP4	0.677	0.097	7.006	0.000
PREP3	0.676	0.091	7.403	0.000
FPRB7	1.171	0.263	4.453	0.000
HELP4	0.639	0.117	5.436	0.000
HELP3	0.502	0.111	4.533	0.000

```
   FPRB6              0.618      0.241      2.568      0.010

 PREP4    WITH
   PREP3              0.412      0.158      2.610      0.009

 HELP2    WITH
   HELP1              1.001      0.074     13.464      0.000
   PREP1              0.389      0.045      8.600      0.000
   PREP2              0.329      0.043      7.727      0.000

 PHPRB2   WITH
   PHPRB1             0.469      0.062      7.514      0.000
   FPRB2             -0.105      0.034     -3.046      0.002
   FPRB9             -0.073      0.019     -3.797      0.000
   FPRB7             -0.223      0.098     -2.268      0.023
   PREP4              0.068      0.031      2.183      0.029
   HELP2             -0.014      0.009     -1.573      0.116
   PREP2              0.016      0.019      0.860      0.390
   FPRB6             -0.124      0.047     -2.651      0.008
   PREP3              0.050      0.031      1.622      0.105
   FPRB1             -0.071      0.034     -2.053      0.040

 FPRB9    WITH
   FPRB8              3.958      0.303     13.082      0.000
   FPRB5              1.714      0.222      7.704      0.000
   PREP2             -0.439      0.080     -5.472      0.000
   PREP1             -0.509      0.083     -6.103      0.000
   FPRB7              2.747      0.336      8.180      0.000
   HELP4              0.750      0.117      6.429      0.000
   HELP3              0.645      0.110      5.854      0.000
   FPRB6              1.462      0.275      5.316      0.000

 FPRB10   WITH
   FPRB9              3.975      0.310     12.801      0.000
   FPRB8              3.275      0.289     11.346      0.000
   HELP3              0.431      0.104      4.130      0.000
   FPRB3              0.515      0.135      3.812      0.000
   FPRB5              1.831      0.273      6.699      0.000
   FPRB4              0.469      0.138      3.409      0.001
   FPRB2              0.397      0.198      2.006      0.045
   FPRB7              3.065      0.393      7.792      0.000
   FPRB6              1.860      0.341      5.458      0.000
   FPRB1              0.443      0.196      2.261      0.024
   HELP4              0.603      0.112      5.385      0.000
   PREP1             -0.374      0.079     -4.714      0.000
   PREP2             -0.288      0.076     -3.790      0.000

 PHPRB5   WITH
   PHPRB4           999.000    999.000    999.000    999.000
   FPRB3              0.767      0.099      7.761      0.000
   FPRB9              1.997      0.181     11.035      0.000
   FPRB4              0.708      0.101      7.035      0.000
   FPRB10             2.102      0.181     11.613      0.000
   FPRB8              1.655      0.179      9.266      0.000
   FPRB5              1.331      0.162      8.236      0.000
   FPRB6              1.200      0.200      6.003      0.000
   FPRB7              1.785      0.223      8.016      0.000
   PREP2             -0.385      0.059     -6.493      0.000
   PREP1             -0.382      0.061     -6.306      0.000
   FPRB1              0.732      0.121      6.070      0.000
   FPRB2              0.691      0.120      5.781      0.000
   HELP4              0.490      0.086      5.698      0.000
   HELP3              0.357      0.081      4.393      0.000
```

```
 PHPRB6   WITH
    PHPRB5             2.221      0.173     12.817      0.000
    FPRB9              2.834      0.225     12.616      0.000
    PHPRB4           999.000    999.000    999.000    999.000
    FPRB2              0.463      0.091      5.113      0.000
    FPRB10             2.157      0.210     10.286      0.000
    FPRB8              2.059      0.215      9.596      0.000
    PREP4              0.745      0.087      8.516      0.000
    PREP3              0.701      0.082      8.531      0.000
    FPRB5              1.112      0.175      6.364      0.000
    FPRB1              0.407      0.090      4.514      0.000
    FPRB6              1.014      0.219      4.621      0.000
    FPRB7              1.622      0.253      6.415      0.000
    HELP4              0.713      0.109      6.526      0.000
    HELP3              0.527      0.103      5.118      0.000
    PHPRB3           999.000    999.000    999.000    999.000

 FPRB7    WITH
    FPRB5              0.929      0.277      3.351      0.001
    FPRB2             -0.012      0.203     -0.059      0.953
    FPRB1              0.039      0.200      0.194      0.846
    PREP2             -0.400      0.173     -2.310      0.021
    PREP1             -0.499      0.174     -2.868      0.004
    HELP3              0.214      0.124      1.726      0.084
    PREP4             -0.650      0.181     -3.589      0.000
    PREP3             -0.585      0.178     -3.293      0.001
    FPRB6              2.211      0.381      5.810      0.000
    HELP4              0.373      0.132      2.813      0.005

 PREP3    WITH
    PREP2              0.083      0.013      6.345      0.000

 PHPRB8   WITH
    PHPRB7             3.126      0.320      9.769      0.000
    PHPRB6             1.777      0.238      7.454      0.000
    FPRB6              1.205      0.277      4.348      0.000
    PHPRB5            -0.154      0.082     -1.877      0.060
    FPRB8              0.970      0.209      4.637      0.000
    FPRB10            -0.464      0.102     -4.567      0.000
    PREP4             -0.387      0.073     -5.267      0.000
    HELP2             -0.049      0.023     -2.117      0.034
    PREP1             -0.275      0.070     -3.923      0.000
    FPRB7             -0.265      0.128     -2.066      0.039
    PREP2             -0.247      0.069     -3.564      0.000
    FPRB5              0.724      0.250      2.894      0.004

 PHPRB9   WITH
    FPRB8              1.088      0.208      5.217      0.000
    PHPRB8             7.881      0.491     16.037      0.000
    PREP3              0.319      0.065      4.887      0.000
    PHPRB7             3.149      0.301     10.446      0.000
    PHPRB6             2.016      0.225      8.976      0.000
    FPRB6              1.477      0.257      5.750      0.000
    FPRB9              0.342      0.099      3.464      0.001
    FPRB5              0.791      0.236      3.359      0.001

 HELP1    WITH
    PREP1              0.410      0.047      8.772      0.000
    PREP2              0.319      0.044      7.303      0.000

 PHPRB3   WITH
    PHPRB2           999.000    999.000    999.000    999.000
    FPRB6            999.000    999.000    999.000    999.000
    FPRB7            999.000    999.000    999.000    999.000
```

```
   FPRB5              999.000    999.000    999.000    999.000
   PREP2              999.000    999.000    999.000    999.000
   PREP1              999.000    999.000    999.000    999.000
   FPRB2              999.000    999.000    999.000    999.000
   FPRB1              999.000    999.000    999.000    999.000

 PHPRB4   WITH
   PREP3              999.000    999.000    999.000    999.000
   PREP4              999.000    999.000    999.000    999.000
   HELP3              999.000    999.000    999.000    999.000
   FPRB8              999.000    999.000    999.000    999.000
   FPRB10             999.000    999.000    999.000    999.000
   FPRB9              999.000    999.000    999.000    999.000
   PHPRB3             999.000    999.000    999.000    999.000
   HELP2              999.000    999.000    999.000    999.000
   PHPRB2             999.000    999.000    999.000    999.000
   PHPRB1             999.000    999.000    999.000    999.000
   FPRB7              999.000    999.000    999.000    999.000
   FPRB6              999.000    999.000    999.000    999.000
   HELP4              999.000    999.000    999.000    999.000
   FPRB2              999.000    999.000    999.000    999.000
   FPRB1              999.000    999.000    999.000    999.000

 FPRB8    WITH
   FPRB5                2.034      0.228      8.933      0.000
   FPRB4               -0.049      0.032     -1.561      0.119
   PREP3               -0.280      0.130     -2.164      0.030
   FPRB7                2.624      0.330      7.948      0.000
   HELP4                0.644      0.118      5.469      0.000
   HELP3                0.598      0.111      5.402      0.000
   FPRB6                1.544      0.269      5.737      0.000
   PREP1               -0.734      0.138     -5.334      0.000
   PREP2               -0.652      0.136     -4.799      0.000
   PREP4               -0.402      0.137     -2.940      0.003

 FPRB5    WITH
   FPRB4                0.559      0.127      4.406      0.000
   HELP3                0.065      0.095      0.684      0.494
   FPRB3                0.476      0.123      3.876      0.000
   PREP3                0.264      0.077      3.441      0.001
   PREP4                0.161      0.086      1.875      0.061
   HELP4                0.237      0.101      2.347      0.019
   PREP1               -0.081      0.037     -2.165      0.030

 PREP2    WITH
   PREP1                0.329      0.133      2.482      0.013

 PHPRB1   WITH
   PREP1               -0.002      0.025     -0.082      0.934
   FPRB7               -0.019      0.090     -0.216      0.829
   PREP2               -0.004      0.027     -0.130      0.897
   HELP3               -0.019      0.014     -1.406      0.160
   FPRB5                0.060      0.033      1.816      0.069

 HELP4    WITH
   HELP2               -0.157      0.061     -2.582      0.010

 HELP3    WITH
   HELP1                0.123      0.060      2.051      0.040

 FPRB6    WITH
   PREP2                0.283      0.089      3.165      0.002
   PREP1                0.188      0.089      2.110      0.035
   HELP3                0.032      0.111      0.286      0.775
```

HELP4	0.115	0.118	0.976	0.329
PREP3	0.129	0.059	2.205	0.027
FPRB5	1.318	0.246	5.347	0.000
**Intercepts**				
PREP1	2.721	0.091	29.771	0.000
PREP2	2.771	0.091	30.444	0.000
PREP3	3.064	0.099	30.940	0.000
PREP4	3.013	0.102	29.667	0.000
HELP1	3.727	0.110	34.013	0.000
HELP2	3.752	0.108	34.609	0.000
HELP3	4.043	0.110	36.727	0.000
HELP4	4.116	0.110	37.482	0.000
FPRB1	2.159	0.084	25.567	0.000
FPRB2	2.180	0.085	25.730	0.000
FPRB3	1.496	0.076	19.773	0.000
FPRB4	1.466	0.077	18.929	0.000
FPRB5	2.677	0.108	24.863	0.000
FPRB6	3.577	0.125	28.726	0.000
FPRB7	2.973	0.138	21.517	0.000
FPRB8	3.375	0.111	30.330	0.000
FPRB9	3.159	0.110	28.708	0.000
FPRB10	2.870	0.107	26.828	0.000
PHPRB1	0.446	0.048	9.310	0.000
PHPRB2	0.445	0.051	8.667	0.000
PHPRB3	0.561	0.051	11.028	0.000
PHPRB4	0.852	0.064	13.370	0.000
PHPRB5	1.436	0.080	17.858	0.000
PHPRB6	2.948	0.098	29.947	0.000
PHPRB7	3.239	0.108	29.890	0.000
PHPRB8	5.775	0.129	44.698	0.000
PHPRB9	5.714	0.122	46.653	0.000
PHPRB10	5.911	0.124	47.594	0.000
**Variances**				
PREP	1.000	0.000	999.000	999.000
FPRB	1.000	0.000	999.000	999.000
**Residual Variances**				
PREP1	0.446	0.135	3.311	0.001
PREP2	0.429	0.133	3.222	0.001
PREP3	0.628	0.155	4.044	0.000
PREP4	0.650	0.165	3.943	0.000
HELP1	1.293	0.098	13.172	0.000
HELP2	0.937	0.091	10.264	0.000
HELP3	0.410	0.074	5.512	0.000
HELP4	0.299	0.079	3.799	0.000
FPRB1	1.186	0.180	6.592	0.000
FPRB2	1.102	0.182	6.070	0.000
FPRB3	1.927	0.133	14.498	0.000
FPRB4	2.045	0.140	14.630	0.000
FPRB5	3.789	0.275	13.754	0.000
FPRB6	4.526	0.371	12.189	0.000
FPRB7	5.394	0.598	9.026	0.000
FPRB8	5.317	0.347	15.323	0.000
FPRB9	4.863	0.324	15.019	0.000
FPRB10	4.955	0.394	12.585	0.000
PHPRB1	0.570	0.061	9.337	0.000
PHPRB2	0.592	0.085	6.995	0.000
PHPRB3	999.000	999.000	999.000	999.000
PHPRB4	999.000	999.000	999.000	999.000
PHPRB5	2.990	0.176	16.957	0.000
PHPRB6	4.915	0.258	19.084	0.000
PHPRB7	6.385	0.327	19.508	0.000

PHPRB8	9.239	0.559	16.526	0.000
PHPRB9	8.299	0.484	17.147	0.000
PHPRB10	8.548	0.499	17.116	0.000
HELP	0.795	0.031	25.299	0.000
PHPRB	0.923	0.024	38.123	0.000

R-SQUARE

Observed Variable	Estimate	S.E.	Est./S.E.	Two-Tailed P-Value
PREP1	0.905	0.029	31.098	0.000
PREP2	0.908	0.029	31.274	0.000
PREP3	0.886	0.029	30.649	0.000
PREP4	0.887	0.029	30.469	0.000
HELP1	0.808	0.017	46.896	0.000
HELP2	0.858	0.015	55.377	0.000
HELP3	0.940	0.012	81.675	0.000
HELP4	0.956	0.012	79.666	0.000
FPRB1	0.703	0.046	15.169	0.000
FPRB2	0.726	0.046	15.667	0.000
FPRB3	0.399	0.038	10.533	0.000
FPRB4	0.391	0.038	10.348	0.000
FPRB5	0.416	0.040	10.288	0.000
FPRB6	0.479	0.042	11.477	0.000
FPRB7	0.495	0.054	9.162	0.000
FPRB8	0.233	0.034	6.797	0.000
FPRB9	0.283	0.035	8.158	0.000
FPRB10	0.227	0.051	4.453	0.000
PHPRB1	0.558	0.048	11.587	0.000
PHPRB2	0.599	0.058	10.291	0.000
PHPRB3	Undefined	0.10482E+01		
PHPRB4	Undefined	0.29691E+01		
PHPRB5	0.174	0.029	5.970	0.000
PHPRB6	0.094	0.021	4.416	0.000
PHPRB7	0.029	0.012	2.472	0.013
PHPRB8	0.012	0.006	1.947	0.051
PHPRB9	0.012	0.006	2.002	0.045
PHPRB10	0.010	0.005	1.906	0.057

Latent Variable	Estimate	S.E.	Est./S.E.	Two-Tailed P-Value
HELP	0.205	0.031	6.505	0.000
PHPRB	0.077	0.024	3.196	0.001

QUALITY OF NUMERICAL RESULTS

Condition Number for the Information Matrix 0.131E-04
(ratio of smallest to largest eigenvalue)

MODEL MODIFICATION INDICES

NOTE: Modification indices for direct effects of observed dependent variables regressed on covariates may not be included. To include these, request MODINDICES (ALL).

Minimum M.I. value for printing the modification index 0.000

	M.I.	E.P.C.	Std E.P.C.	StdYX E.P.C.

BY Statements

PREP	BY HELP1	0.015	-0.002	-0.003	-0.001
PREP	BY HELP2	0.794	-0.012	-0.024	-0.009
PREP	BY HELP3	0.838	0.018	0.036	0.014
PREP	BY HELP4	0.244	0.010	0.020	0.008
PREP	BY FPRB1	0.039	0.002	0.004	0.002
PREP	BY FPRB2	1.490	-0.011	-0.023	-0.011
PREP	BY FPRB3	2.677	-0.018	-0.037	-0.021
PREP	BY FPRB4	1.841	0.015	0.032	0.017
PREP	BY FPRB5	0.995	-0.037	-0.076	-0.030
PREP	BY FPRB6	6.798	0.111	0.229	0.078
PREP	BY FPRB7	8.553	0.263	0.542	0.166
PREP	BY FPRB8	12.813	-0.213	-0.438	-0.166
PREP	BY FPRB9	12.250	0.087	0.178	0.068
PREP	BY FPRB10	1.140	-0.027	-0.056	-0.022
PREP	BY PHPRB1	0.003	0.001	0.001	0.001
PREP	BY PHPRB2	0.151	-0.005	-0.009	-0.008
PREP	BY PHPRB3	0.057	-0.003	-0.007	-0.006
PREP	BY PHPRB4	0.172	0.016	0.033	0.022
PREP	BY PHPRB5	0.024	0.004	0.009	0.005
PREP	BY PHPRB6	0.250	0.012	0.024	0.010
PREP	BY PHPRB7	5.376	0.065	0.134	0.052
PREP	BY PHPRB8	0.139	-0.011	-0.023	-0.007
PREP	BY PHPRB9	13.068	0.081	0.166	0.057
PREP	BY PHPRB10	0.881	-0.023	-0.047	-0.016
HELP	BY PREP1	0.008	-0.001	-0.002	-0.001
HELP	BY PREP2	0.006	0.001	0.002	0.001
HELP	BY PREP3	0.000	0.000	0.000	0.000
HELP	BY PREP4	0.000	0.000	0.000	0.000
HELP	BY FPRB1	0.314	0.005	0.012	0.006
HELP	BY FPRB2	1.033	-0.009	-0.021	-0.011
HELP	BY FPRB3	1.000	-0.011	-0.025	-0.014
HELP	BY FPRB4	0.101	-0.003	-0.008	-0.004
HELP	BY FPRB5	0.016	0.005	0.011	0.004
HELP	BY FPRB6	4.432	0.084	0.195	0.066
HELP	BY FPRB7	8.544	0.126	0.292	0.089
HELP	BY FPRB8	12.781	-0.101	-0.236	-0.090
HELP	BY FPRB9	7.399	0.062	0.143	0.055
HELP	BY FPRB10	1.988	-0.050	-0.116	-0.046
HELP	BY PHPRB1	0.311	0.004	0.010	0.009
HELP	BY PHPRB2	0.513	-0.006	-0.014	-0.011
HELP	BY PHPRB3	4.415	-0.025	-0.059	-0.049
HELP	BY PHPRB4	4.527	0.074	0.172	0.114
HELP	BY PHPRB5	0.116	0.008	0.020	0.010
HELP	BY PHPRB6	2.888	0.035	0.081	0.035
HELP	BY PHPRB7	2.301	0.039	0.090	0.035
HELP	BY PHPRB8	2.560	-0.036	-0.083	-0.027
HELP	BY PHPRB9	1.662	0.024	0.056	0.019
HELP	BY PHPRB10	2.315	0.032	0.075	0.026
FPRB	BY PREP1	0.017	-0.002	-0.003	-0.001
FPRB	BY PREP2	0.013	0.001	0.002	0.001
FPRB	BY PREP3	0.001	0.000	0.001	0.000
FPRB	BY PREP4	0.000	0.000	0.000	0.000
FPRB	BY HELP1	0.174	0.006	0.010	0.004
FPRB	BY HELP2	0.755	0.013	0.021	0.008
FPRB	BY HELP3	2.897	-0.042	-0.070	-0.027
FPRB	BY HELP4	0.107	-0.008	-0.014	-0.005
FPRB	BY PHPRB1	0.060	0.003	0.006	0.005
FPRB	BY PHPRB2	0.466	-0.010	-0.017	-0.014
FPRB	BY PHPRB3	0.939	-0.026	-0.044	-0.036
FPRB	BY PHPRB4	2.300	0.188	0.316	0.209
FPRB	BY PHPRB5	0.101	0.027	0.046	0.024
FPRB	BY PHPRB6	6.418	0.113	0.189	0.081

FPRB	BY PHPRB7	0.143	0.018	0.029	0.011
FPRB	BY PHPRB8	0.464	-0.025	-0.042	-0.014
FPRB	BY PHPRB9	0.125	0.011	0.018	0.006
FPRB	BY PHPRB10	1.200	0.040	0.067	0.023
PHPRB	BY PREP1	0.012	-0.001	-0.001	-0.001
PHPRB	BY PREP2	0.006	0.001	0.001	0.000
PHPRB	BY PREP3	0.916	-0.029	-0.024	-0.010
PHPRB	BY PREP4	1.080	0.032	0.027	0.011
PHPRB	BY HELP1	2.054	-0.031	-0.027	-0.010
PHPRB	BY HELP2	2.254	0.033	0.028	0.011
PHPRB	BY HELP3	0.326	-0.021	-0.018	-0.007
PHPRB	BY HELP4	0.199	0.017	0.015	0.006
PHPRB	BY FPRB1	0.210	0.014	0.012	0.006
PHPRB	BY FPRB2	0.954	-0.030	-0.026	-0.013
PHPRB	BY FPRB3	0.256	-0.008	-0.006	-0.004
PHPRB	BY FPRB4	0.674	0.013	0.011	0.006
PHPRB	BY FPRB5	0.033	0.011	0.010	0.004
PHPRB	BY FPRB6	0.615	0.113	0.096	0.032
PHPRB	BY FPRB7	2.923	-1.584	-1.342	-0.410
PHPRB	BY FPRB8	0.481	0.049	0.042	0.016
PHPRB	BY FPRB9	2.026	-0.082	-0.069	-0.027
PHPRB	BY FPRB10	0.928	0.072	0.061	0.024

WITH Statements

PREP3	WITH PREP1	0.000	0.001	0.001	0.001
PREP4	WITH PREP1	0.000	0.002	0.002	0.003
PREP4	WITH PREP2	0.001	-0.002	-0.002	-0.004
HELP1	WITH PREP3	0.377	0.008	0.008	0.009
HELP1	WITH PREP4	0.386	-0.008	-0.008	-0.009
HELP2	WITH PREP3	0.416	-0.008	-0.008	-0.010
HELP2	WITH PREP4	0.215	0.006	0.006	0.007
HELP3	WITH PREP1	0.789	-0.014	-0.014	-0.032
HELP3	WITH PREP2	0.636	0.012	0.012	0.027
HELP3	WITH PREP3	0.001	-0.001	-0.001	-0.001
HELP3	WITH PREP4	0.064	0.005	0.005	0.010
HELP3	WITH HELP2	0.039	0.026	0.026	0.043
HELP4	WITH PREP1	0.737	0.013	0.013	0.036
HELP4	WITH PREP2	0.599	-0.011	-0.011	-0.031
HELP4	WITH PREP3	0.016	0.002	0.002	0.006
HELP4	WITH PREP4	0.004	-0.001	-0.001	-0.003
HELP4	WITH HELP1	0.256	-0.066	-0.066	-0.107
HELP4	WITH HELP3	0.154	0.070	0.070	0.200
FPRB1	WITH PREP1	0.159	0.003	0.003	0.005
FPRB1	WITH PREP2	0.000	0.000	0.000	0.000
FPRB1	WITH PREP3	0.011	0.001	0.001	0.001
FPRB1	WITH PREP4	0.424	-0.008	-0.008	-0.009
FPRB1	WITH HELP1	1.178	0.031	0.031	0.025
FPRB1	WITH HELP2	0.836	0.015	0.015	0.014
FPRB1	WITH HELP3	0.121	-0.005	-0.005	-0.007
FPRB1	WITH HELP4	0.590	-0.012	-0.012	-0.020
FPRB2	WITH PREP1	0.199	-0.004	-0.004	-0.005
FPRB2	WITH PREP2	0.060	0.002	0.002	0.003
FPRB2	WITH PREP3	0.006	-0.001	-0.001	-0.001
FPRB2	WITH PREP4	0.003	0.001	0.001	0.001
FPRB2	WITH HELP2	0.678	-0.016	-0.016	-0.015
FPRB2	WITH HELP3	0.003	-0.001	-0.001	-0.001
FPRB2	WITH HELP4	0.255	0.008	0.008	0.013
FPRB3	WITH PREP1	0.063	0.002	0.002	0.003
FPRB3	WITH PREP2	0.457	-0.007	-0.007	-0.007
FPRB3	WITH PREP3	0.171	0.014	0.014	0.013
FPRB3	WITH PREP4	0.002	-0.001	-0.001	-0.001
FPRB3	WITH HELP1	0.161	-0.004	-0.004	-0.003
FPRB3	WITH HELP2	0.106	0.003	0.003	0.002

FPRB3	WITH HELP3	0.069	-0.004	-0.004	-0.005
FPRB3	WITH HELP4	0.074	0.005	0.005	0.006
FPRB3	WITH FPRB1	0.001	0.000	0.000	0.000
FPRB3	WITH FPRB2	0.000	0.000	0.000	0.000
FPRB4	WITH PREP1	0.030	-0.002	-0.002	-0.002
FPRB4	WITH PREP2	0.141	0.004	0.004	0.004
FPRB4	WITH PREP4	0.196	0.009	0.009	0.008
FPRB4	WITH HELP1	0.015	-0.001	-0.001	-0.001
FPRB4	WITH HELP2	0.124	-0.004	-0.004	-0.003
FPRB4	WITH HELP3	0.117	0.006	0.006	0.006
FPRB4	WITH HELP4	0.000	0.000	0.000	0.000
FPRB4	WITH FPRB1	0.498	-0.007	-0.007	-0.005
FPRB4	WITH FPRB2	0.721	0.009	0.009	0.006
FPRB5	WITH PREP2	1.568	-0.175	-0.175	-0.137
FPRB5	WITH HELP1	0.154	0.013	0.013	0.006
FPRB5	WITH HELP2	0.047	-0.007	-0.007	-0.004
FPRB5	WITH FPRB1	0.212	0.015	0.015	0.007
FPRB5	WITH FPRB2	0.326	-0.019	-0.019	-0.009
FPRB6	WITH PREP4	3.641	0.355	0.355	0.207
FPRB6	WITH HELP1	0.035	-0.007	-0.007	-0.003
FPRB6	WITH HELP2	0.155	0.014	0.014	0.007
FPRB6	WITH FPRB1	0.006	-0.003	-0.003	-0.001
FPRB6	WITH FPRB2	0.052	-0.009	-0.009	-0.004
FPRB6	WITH FPRB3	0.088	0.013	0.013	0.004
FPRB6	WITH FPRB4	0.011	-0.004	-0.004	-0.001
FPRB7	WITH HELP1	0.051	0.008	0.008	0.003
FPRB7	WITH HELP2	0.093	0.011	0.011	0.005
FPRB7	WITH FPRB3	0.269	-0.022	-0.022	-0.007
FPRB7	WITH FPRB4	0.001	0.001	0.001	0.000
FPRB8	WITH HELP1	0.028	0.004	0.004	0.002
FPRB8	WITH HELP2	0.724	-0.022	-0.022	-0.010
FPRB8	WITH FPRB1	0.402	0.016	0.016	0.006
FPRB8	WITH FPRB2	0.195	-0.011	-0.011	-0.005
FPRB8	WITH FPRB3	0.550	0.065	0.065	0.020
FPRB9	WITH PREP3	0.016	0.004	0.004	0.003
FPRB9	WITH PREP4	0.528	0.026	0.026	0.015
FPRB9	WITH HELP1	0.151	0.008	0.008	0.003
FPRB9	WITH HELP2	0.017	-0.002	-0.002	-0.001
FPRB9	WITH FPRB1	0.007	0.002	0.002	0.001
FPRB9	WITH FPRB2	0.017	-0.003	-0.003	-0.001
FPRB9	WITH FPRB3	0.172	0.014	0.014	0.005
FPRB9	WITH FPRB4	0.186	-0.016	-0.016	-0.005
FPRB10	WITH PREP3	0.056	-0.008	-0.008	-0.004
FPRB10	WITH PREP4	0.000	0.001	0.001	0.000
FPRB10	WITH HELP1	0.175	-0.010	-0.010	-0.004
FPRB10	WITH HELP2	0.086	0.007	0.007	0.003
PHPRB1	WITH PREP3	0.198	-0.009	-0.009	-0.014
PHPRB1	WITH PREP4	0.091	0.006	0.006	0.010
PHPRB1	WITH HELP1	0.120	-0.004	-0.004	-0.005
PHPRB1	WITH HELP2	0.400	0.009	0.009	0.012
PHPRB1	WITH HELP4	0.002	-0.001	-0.001	-0.002
PHPRB1	WITH FPRB1	0.001	-0.001	-0.001	-0.001
PHPRB1	WITH FPRB2	0.869	-0.019	-0.019	-0.025
PHPRB1	WITH FPRB3	0.580	-0.007	-0.007	-0.007
PHPRB1	WITH FPRB4	1.336	0.012	0.012	0.011
PHPRB1	WITH FPRB6	0.802	0.086	0.086	0.053
PHPRB1	WITH FPRB8	0.009	0.003	0.003	0.002
PHPRB1	WITH FPRB9	0.006	0.002	0.002	0.001
PHPRB1	WITH FPRB10	0.009	0.003	0.003	0.002
PHPRB2	WITH PREP1	0.002	-0.002	-0.002	-0.004
PHPRB2	WITH HELP1	0.000	0.000	0.000	0.000
PHPRB2	WITH HELP3	1.439	-0.029	-0.029	-0.058
PHPRB2	WITH HELP4	0.488	0.013	0.013	0.031
PHPRB2	WITH FPRB3	0.733	0.009	0.009	0.008

PHPRB2	WITH FPRB4	0.750	-0.009	-0.009	-0.008
PHPRB2	WITH FPRB5	0.043	0.016	0.016	0.011
PHPRB2	WITH FPRB8	0.036	0.006	0.006	0.003
PHPRB2	WITH FPRB10	0.584	-0.023	-0.023	-0.014
PHPRB3	WITH PREP3	0.001	-0.001	-0.001	999.000
PHPRB3	WITH PREP4	0.124	0.007	0.007	999.000
PHPRB3	WITH HELP1	1.164	-0.016	-0.016	999.000
PHPRB3	WITH HELP2	0.020	0.002	0.002	999.000
PHPRB3	WITH HELP3	0.641	0.019	0.019	999.000
PHPRB3	WITH HELP4	0.097	-0.008	-0.008	999.000
PHPRB3	WITH FPRB3	0.111	-0.005	-0.005	999.000
PHPRB3	WITH FPRB4	0.182	0.006	0.006	999.000
PHPRB3	WITH FPRB8	0.007	0.004	0.004	999.000
PHPRB3	WITH FPRB9	0.690	-0.029	-0.029	999.000
PHPRB3	WITH FPRB10	1.899	0.057	0.057	999.000
PHPRB3	WITH PHPRB1	2.861	0.678	0.678	999.000
PHPRB4	WITH PREP1	0.035	0.004	0.004	999.000
PHPRB4	WITH PREP2	0.089	-0.007	-0.007	999.000
PHPRB4	WITH HELP1	4.616	0.326	0.326	999.000
PHPRB4	WITH FPRB3	0.047	-0.005	-0.005	999.000
PHPRB4	WITH FPRB4	0.010	0.002	0.002	999.000
PHPRB4	WITH FPRB5	0.003	0.006	0.006	999.000
PHPRB5	WITH PREP3	0.888	-0.038	-0.038	-0.027
PHPRB5	WITH PREP4	0.896	0.039	0.039	0.028
PHPRB5	WITH HELP1	0.050	0.006	0.006	0.003
PHPRB5	WITH HELP2	0.024	-0.004	-0.004	-0.002
PHPRB5	WITH PHPRB1	0.162	0.011	0.011	0.008
PHPRB5	WITH PHPRB2	0.006	-0.002	-0.002	-0.002
PHPRB5	WITH PHPRB3	0.273	-0.042	-0.042	999.000
PHPRB6	WITH PREP1	1.177	-0.025	-0.025	-0.017
PHPRB6	WITH PREP2	1.137	0.025	0.025	0.017
PHPRB6	WITH HELP1	0.886	-0.020	-0.020	-0.008
PHPRB6	WITH HELP2	1.305	0.023	0.023	0.011
PHPRB6	WITH FPRB3	0.004	-0.002	-0.002	-0.001
PHPRB6	WITH FPRB4	0.459	0.019	0.019	0.006
PHPRB6	WITH PHPRB1	0.425	-0.020	-0.020	-0.012
PHPRB6	WITH PHPRB2	0.101	0.011	0.011	0.006
PHPRB7	WITH PREP1	0.144	0.010	0.010	0.006
PHPRB7	WITH PREP2	0.006	0.002	0.002	0.001
PHPRB7	WITH HELP1	0.449	0.017	0.017	0.006
PHPRB7	WITH HELP2	0.311	-0.014	-0.014	-0.006
PHPRB7	WITH FPRB1	1.090	0.040	0.040	0.014
PHPRB7	WITH FPRB2	0.048	0.008	0.008	0.003
PHPRB7	WITH FPRB3	0.568	-0.023	-0.023	-0.006
PHPRB7	WITH FPRB4	0.463	-0.021	-0.021	-0.006
PHPRB7	WITH PHPRB2	0.234	0.028	0.028	0.014
PHPRB7	WITH PHPRB3	0.255	-0.038	-0.038	999.000
PHPRB8	WITH PREP3	0.387	0.080	0.080	0.033
PHPRB8	WITH HELP1	0.002	-0.003	-0.003	-0.001
PHPRB8	WITH HELP3	0.397	-0.026	-0.026	-0.013
PHPRB8	WITH HELP4	0.004	0.003	0.003	0.002
PHPRB8	WITH FPRB1	0.076	-0.007	-0.007	-0.002
PHPRB8	WITH FPRB2	0.098	0.008	0.008	0.002
PHPRB8	WITH FPRB3	0.310	-0.015	-0.015	-0.004
PHPRB8	WITH FPRB4	0.132	0.010	0.010	0.002
PHPRB8	WITH FPRB9	1.020	0.184	0.184	0.027
PHPRB8	WITH PHPRB1	0.174	0.010	0.010	0.004
PHPRB8	WITH PHPRB2	0.237	-0.012	-0.012	-0.005
PHPRB8	WITH PHPRB3	0.048	0.008	0.008	999.000
PHPRB8	WITH PHPRB4	0.004	-0.006	-0.006	999.000
PHPRB9	WITH PREP1	0.037	0.004	0.004	0.002
PHPRB9	WITH PREP2	0.064	0.006	0.006	0.003
PHPRB9	WITH PREP4	0.905	0.046	0.046	0.020
PHPRB9	WITH HELP1	0.222	-0.011	-0.011	-0.003

PHPRB9	WITH HELP2	0.001	0.001	0.001	0.000
PHPRB9	WITH HELP3	0.160	0.014	0.014	0.008
PHPRB9	WITH HELP4	0.001	0.002	0.002	0.001
PHPRB9	WITH FPRB1	0.122	-0.008	-0.008	-0.003
PHPRB9	WITH FPRB2	0.037	0.004	0.004	0.001
PHPRB9	WITH FPRB3	0.024	0.004	0.004	0.001
PHPRB9	WITH FPRB4	0.218	0.011	0.011	0.003
PHPRB9	WITH FPRB7	0.364	-0.066	-0.066	-0.010
PHPRB9	WITH FPRB10	0.325	0.047	0.047	0.007
PHPRB9	WITH PHPRB1	0.034	-0.004	-0.004	-0.002
PHPRB9	WITH PHPRB2	0.332	0.013	0.013	0.006
PHPRB9	WITH PHPRB3	1.331	-0.039	-0.039	999.000
PHPRB9	WITH PHPRB4	0.234	0.041	0.041	999.000
PHPRB9	WITH PHPRB5	1.032	0.076	0.076	0.015
PHPRB10	WITH PREP1	0.000	0.000	0.000	0.000
PHPRB10	WITH PREP2	0.532	-0.017	-0.017	-0.009
PHPRB10	WITH PREP4	0.271	0.026	0.026	0.011
PHPRB10	WITH HELP1	0.052	0.005	0.005	0.002
PHPRB10	WITH HELP2	0.000	0.000	0.000	0.000
PHPRB10	WITH HELP3	0.338	0.031	0.031	0.016
PHPRB10	WITH FPRB3	0.088	-0.008	-0.008	-0.002
PHPRB10	WITH FPRB4	0.290	-0.015	-0.015	-0.004
PHPRB10	WITH FPRB7	1.353	0.131	0.131	0.019
PHPRB10	WITH FPRB10	0.559	0.064	0.064	0.010
PHPRB10	WITH PHPRB1	0.010	0.004	0.004	0.002
PHPRB10	WITH PHPRB4	0.007	-0.010	-0.010	999.000
PHPRB10	WITH PHPRB5	0.215	-0.035	-0.035	-0.007

DIAGRAM INFORMATION

Use View Diagram under the Diagram menu in the Mplus Editor to view the diagram.
If running Mplus from the Mplus Diagrammer, the diagram opens automatically.

Diagram output
d:\flood21ok.dgm

Beginning Time: 19:59:01
Ending Time: 19:59:01
Elapsed Time: 00:00:00

MUTHEN & MUTHEN
3463 Stoner Ave.
Los Angeles, CA 90066

Tel: (310) 391-9971
Fax: (310) 391-8971
Web: www.StatModel.com
Support: Support@StatModel.com

Copyright (c) 1998-2015 Muthen & Muthen
